# Gendered experiences of people living with tuberculosis or leprosy: A global systematic review

**DOI:** 10.1371/journal.pntd.0014711

**Published:** 2026-09-08

**Authors:** Sophie C. W. Unterkircher, Juliana Lenz, Grace Mwasuka

**Affiliations:** 1 Department of Global Health, Institute of Public Health and Nursing Research, University of Bremen, Bremen, Germany; 2 German Leprosy and Tuberculosis Relief Association Tanzania (GLRA), Dar es Salaam, Tanzania; Marie Adelaide Leprosy Center, PAKISTAN

## Abstract

**Background:**

Classified as poverty-related diseases (PRD) leprosy and tuberculosis (TB) are long-standing mycobacterial diseases strongly linked to poverty, stigma, and social exclusion, disproportionately affecting vulnerable populations. Gender norms further shape how individuals experience illness, access to care, and coping strategies, but evidence remains fragmented and largely disease-specific. This systematic review synthesizes qualitative evidence on lived experiences of people affected by TB or leprosy worldwide and compares gender inequities across both diseases to identify shared social and structural determinants.

**Methods/principal findings:**

A systematic review following PRISMA guidelines searched PubMed, Embase, Scopus, and Web of Science for peer-reviewed qualitative and mixed methods studies (1995–08/2025) reporting primary qualitative data on gendered experiences of people affected by TB or leprosy. Studies in all countries and languages were eligible. Screening and extraction were conducted using *Rayyan.* Inductive thematic synthesis was performed using *MAXQDA*. Quality was assessed using the CASP checklist. A total of 3204 records were identified, with 57 studies from 29 countries across five continents included. Across settings, women experienced greater barriers to diagnosis and care, more severe marital and familial consequences, higher stigma and social exclusion, and greater psychosocial burden than men. Men’s experiences were shaped by norms of masculinity, particularly regarding economic provision and employment. Despite disease differences, psychosocial and socioeconomic impacts were similar across TB and leprosy, embedded in gendered social norms, power relations, and social determinants of health.

**Conclusions/significance:**

Gender-based inequities are pervasive across TB and leprosy globally. Comparing both diseases reveals shared social mechanisms beyond biomedical differences. Addressing them requires moving beyond biomedical models and integrating gender as a structural determinant of health. Gender-sensitive, participatory, and intersectional interventions are needed to improve early diagnosis, treatment adherence, and psychosocial and socioeconomic outcomes.

**Registration:**

Registered on Open Science Framework (OSF) on 01/09/2025.

## Author summary

Tuberculosis and leprosy are infectious diseases that remain closely linked to poverty, stigma, and social inequality. In many countries, health services are increasingly combining the two diseases in their control programs because they affect similar populations and share overlapping social and economic drivers. Studying them together can therefore provide useful insights for improving care. In particular, comparing leprosy with tuberculosis, which receives far greater global attention and funding, can help make the often overlooked social and psychological burdens of leprosy more visible. In this study, we reviewed qualitative research from around the world to understand how gender shapes the lived experiences of people affected by tuberculosis and leprosy. We included 57 studies from 29 countries and examined experiences related to seeking care, living with disease, and coping with social consequences. We found that women consistently face greater barriers to diagnosis and treatment, higher stigma, and more severe impacts on family and social relationships. Men’s experiences were strongly influenced by expectations to provide financially and to appear strong and self-reliant. Despite differences between the diseases, the social and psychological consequences were remarkably similar, suggesting they are driven mainly by gender norms and social inequalities rather than medical factors alone.

## Introduction

As two of the most prevalent and long-standing mycobacterial infections, leprosy and tuberculosis (TB) continue to pose a significant global health challenge [1]. While TB remains one of the leading causes of death, with around 11 million new cases reported each year, leprosy has been classified as a neglected tropical disease (NTD) since 2003, with around 200 000 new cases reported annually [2–4]. Leprosy is caused by the bacterium *Mycobacterium leprae* and is spread through prolonged close contact with an untreated person affected by the disease, via droplets from the nose and mouth [4]. The chronic disease mainly affects the peripheral nerves and skin; however, if left untreated, it may cause permanent disabilities [4]. TB is caused by *Mycobacterium tuberculosis*, which belongs to the same genus as the bacterium causing leprosy [5]. It is transmitted through the inhalation of airborne droplets from a person with active pulmonary TB who coughs, speaks, sings or sneezes and is thus far more contagious than leprosy [5]. TB is classified as a multisystem disease, although it primarily affects the lungs [1,5]. Furthermore, both diseases are marked by granulomatous inflammatory reactions, and the clinical presentation depends on the patient’s immune response [4,5]. In addition to sharing etiopathogenic similarities, both diseases are strongly shaped by social determinants of health (SDH) that intersect to influence susceptibility and disease experiences [6,7]. These include socioeconomic risk factors, such as social vulnerability, poverty, limited access to healthcare, larger household sizes and low literacy [8–10]. As part of the broader group of poverty-related diseases (PRD), which disproportionately affect low-and middle-income countries (LMIC), it is essential to understand how these non-medical factors influence disease experiences [5–7,11]. These intersecting SDH also contribute to the persistence of stigma, which has long been associated with both leprosy and TB, despite their epidemiological differences in terms of global case prevalence and modes of transmission [12,13]. As a socially mediated process, stigma shapes the lives of those affected, reinforces marginalization, exacerbating existing inequalities [14,15]. Among these determinants gender represents a key axis through which various SDH intersect. Gender norms and roles influence how individuals experience illness, with consequences for social expectations, family dynamics and economic wellbeing [16,17]. Studies have shown that underlying gender roles and norms often influence care-seeking behavior, treatment adherence and mental health [14,15,18,19], while biological sex-related aspects such as life expectancy, immune responses and metabolic differences also play a role [20,21].

Gender refers to the socially constructed norms, roles and behaviors associated with being a woman, man, or person of a diverse gender identity. These norms vary across societies and over time. As gender is hierarchical and intersects with other social and economic inequalities, it profoundly influences individual’s exposure to stigma, access to healthcare and overall experience of living with TB or leprosy [22]. Worldwide, and particularly in many LMIC traditional gender roles tend to assign caregiving and domestic responsibilities to women, while positioning men as the primary economic breadwinners with greater decision-making power and mobility [22–24]. These dynamics influence how individuals recognize symptoms, seek care, adhere to treatment and navigate social relationships during illness [14,15,18,19]. Recognizing these patterns is essential for understanding the lived experiences of those affected by TB or leprosy, and for ensuring that interventions acknowledge, adapt to, and address gendered barriers.

Current evidence remains fragmented; although gendered experiences have been well documented, reviews often focus on one disease, within one setting, or without explicitly analyzing gender as a central theme. This is a critical gap, given that although TB and leprosy differ in their epidemiology, transmission dynamics, and global health impact, both are classified as PRD and are shaped strongly by overlapping social and structural determinants of health [8–10]. This shared ground within broader socioeconomic and health system constraints provides a justified basis for a shared qualitative synthesis focused on lived experiences. The aim of this review is therefore not to compare clinical disease characteristics, but to examine how gender shapes experiences across two conditions that are in part socially produced and structurally influenced. Furthermore, this study aims to give a global platform to the voices of those directly affected.

This review uses the terms “men” and “women” as reported in the include studies, which predominantly classified participants in binary terms. People with diverse gender identities were not involved. These terms are therefore used to reflect how gender was operationalized in the primary literature and to describe reported lived experiences as documented, without assuming gender identity beyond what was reported. Accordingly, “men” and “women” are used to refer to gendered social roles. Where biological or epidemiological differences are discussed, these are explicitly framed as sex-based (biomedical) factors.

## Methods

### Search strategy

A systematic review was conducted, following the PRISMA guidelines (see PRISMA Checklist). A search strategy (see S1 File, Search Strategy) was created in collaboration with an information science expert from the University of Bremen. After piloting the pre-designed search strings, we searched *PubMed*, *Embase*, *Scopus* and *Web of Science* for eligible papers. The final search took place on 04/08/2025. Evidence on the lived experiences of people affected by leprosy or TB was collected from the global literature.

### Eligibility criteria

Studies were included or excluded based on the defined criteria detailed in the table below (see Table 1).

**Table 1:** Inclusion and exclusion criteria.

Topic	Inclusion	Exclusion
**Study Characteristics**	• Qualitative study • Mixed-methods with detailed qualitative findings • Primary data • Published ≥1995 • Peer-reviewed article • All languages • All countries	• Quantitative only • No primary data • Published before 1995 • Grey literature
**Population**	• Persons Affected by TB or Leprosy • Any age, gender, or location • TB or Leprosy as main focus	• Not about TB or Leprosy • Not about people affected • TB/Leprosy included but primary focus on other diseases
**Themes**	• Lived experiences (psychosocial) • Gender-related themes	• No gender-related themes • No lived experiences (psychosocial)

### Data collection

All studies identified by the final search on 04/08/2025 were automatically screened for duplicates using Rayyan software, after which SCWU manually checked and excluded any suggested duplicates. An initial pilot title/abstract screening was performed by SCWU, JL and the first author’s doctoral supervisor Melanie Böckmann (MB) for the first fifty studies to ensure consistency in eligibility criteria. SCWU, JL, and MB then proceeded to screen titles and abstracts independently for all studies. Seven corresponding authors were contacted with request for full-text-access of whom two responded. Six Portuguese studies and one Spanish study were translated into English using DeepL Premium software. SCWU and JL then proceeded to conduct full-text screening. Between deduplication, title/abstract, and full-text screening, SCWU, JL, and MB met continuously to discuss conflicts and uncertainties in the screening process, reaching consensus before moving on to the next step. The screening took place on the Rayyan software platform in a blinded format.

### Data extraction and analysis

Following study selection, SCWU led the data analysis phase, with JL and MB contributing to early-stage interpretive review and providing feedback during initial coding development. The included studies were extracted using an extraction sheet created in Rayyan by SCWU. Each study was assigned an ID, and the following categories were used for extraction: title, year of publication, authors, study design, disease, country/region, gender, demographics, and lived experience themes (see S2 File, Data Extraction).

The analysis was guided by Braun and Clarke’s six-phase reflexive thematic analysis framework [25], following a data driven, inductive coding approach capturing the lived experiences by persons affected that arose from the included evidence. First, all included studies were re-read in full by SCWU to ensure familiarization with the extracted data prior to coding. Second, line-by-line coding was conducted by SCWU using the qualitative data analysis software MAXQDA 20. Third, an initial coding scheme was developed. Codes were examined for patterns of meaning and grouped into preliminary categories reflecting shared concepts across studies. Fourth, the coding scheme was reviewed by JL and MB, who provided feedback on the coding structure and interpretive consistency. Any discrepancies in interpretation were resolved through discussion and consensus. Codes were then refined inductively, sub-categories were created and applied systematically across all included studies by SCWU. Codes were iteratively compared, merged and reorganized. Fifth, categories were developed into overarching analytical themes representing patterns in lived experiences across the included literature. The final coding framework can be found in the S5 File. The evolving analytical framework was continuously examined for overlap and consistency between categories by SCWU and reviewed by JL and MB. Finally, themes were summarized in an Excel table for each study, differentiating between gender and disease (see S3 File, Synthesis of Study Results).

Furthermore, quotations from participants were included in the manuscript and purposively selected to: amplify participants’ voices, enhance transparency by demonstrating how interpretations were grounded in the primary data, and provide clear and illustrative examples of the identified themes. As this review draws on secondary data, the quotations are excerpts that were selected by the original study authors. In the instance that multiple quotations were available for a theme, the most conceptually representative or illustrative ones were prioritized. Efforts were made to include quotations reflecting a range of study contexts and participant perspectives. Quotations are presented as illustrative examples and do not imply frequency, unless explicitly stated.

### Data appraisal and risk of bias

The selected studies were appraised for their quality by SCWU. The CASP quality appraisal checklist was used for qualitative studies, including the qualitative components of mixed- method studies [26]. Study quality was not used as a basis for exclusion or weighting, as the majority of studies were judged to be of good quality. However, the appraisal informed a cautious interpretation of the findings, particularly where author reflexivity, analytical rigor, or study limitations were not adequately reported. Quality appraisal results can be found in the S4 File.

### Registration

The Systematic Review was registered on Open Science Framework (OSF) on 01/09/2025.

## Results

### Demographics and study population

**Fig 1:**
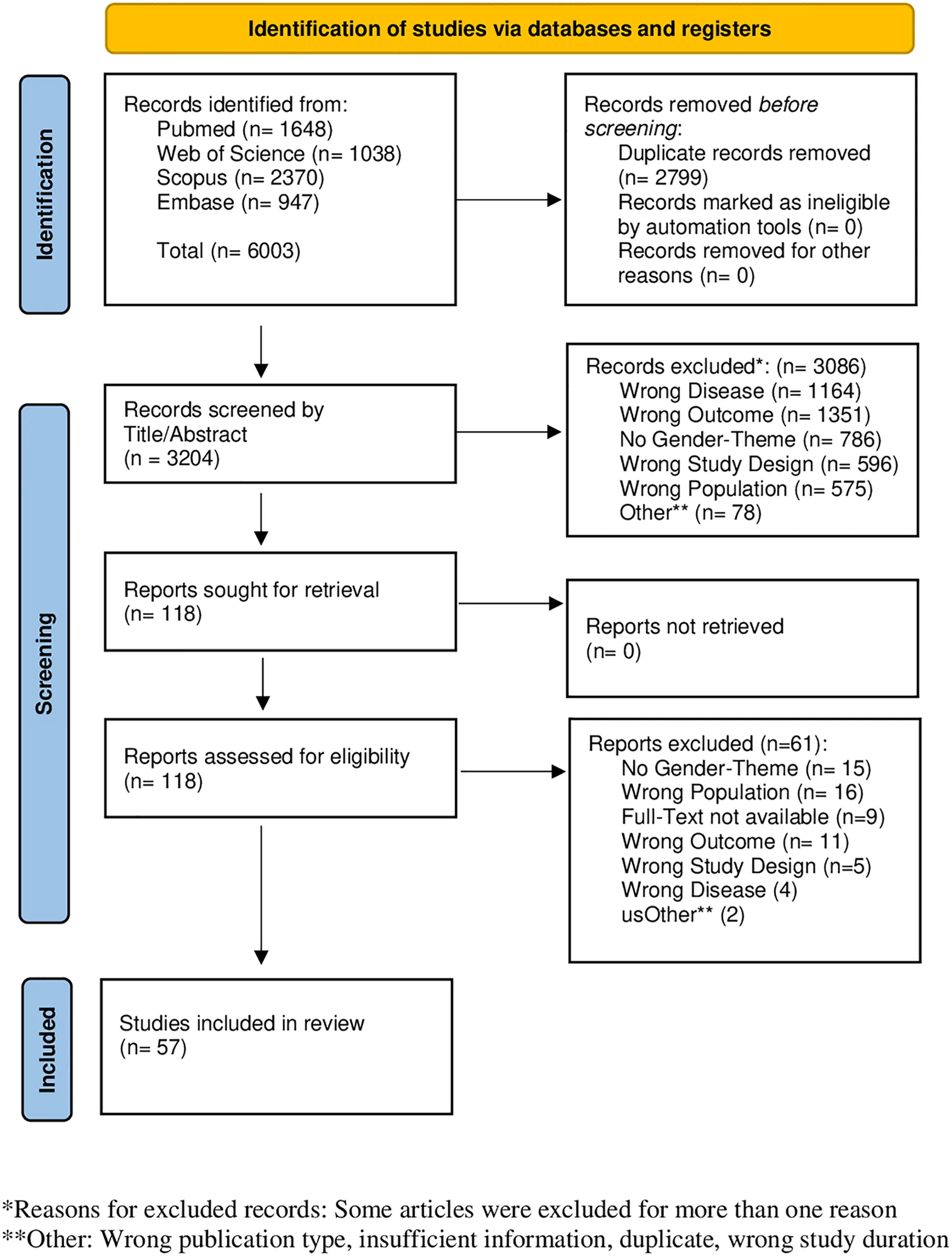
PRISMA Flow Diagram.

**Fig 2:**
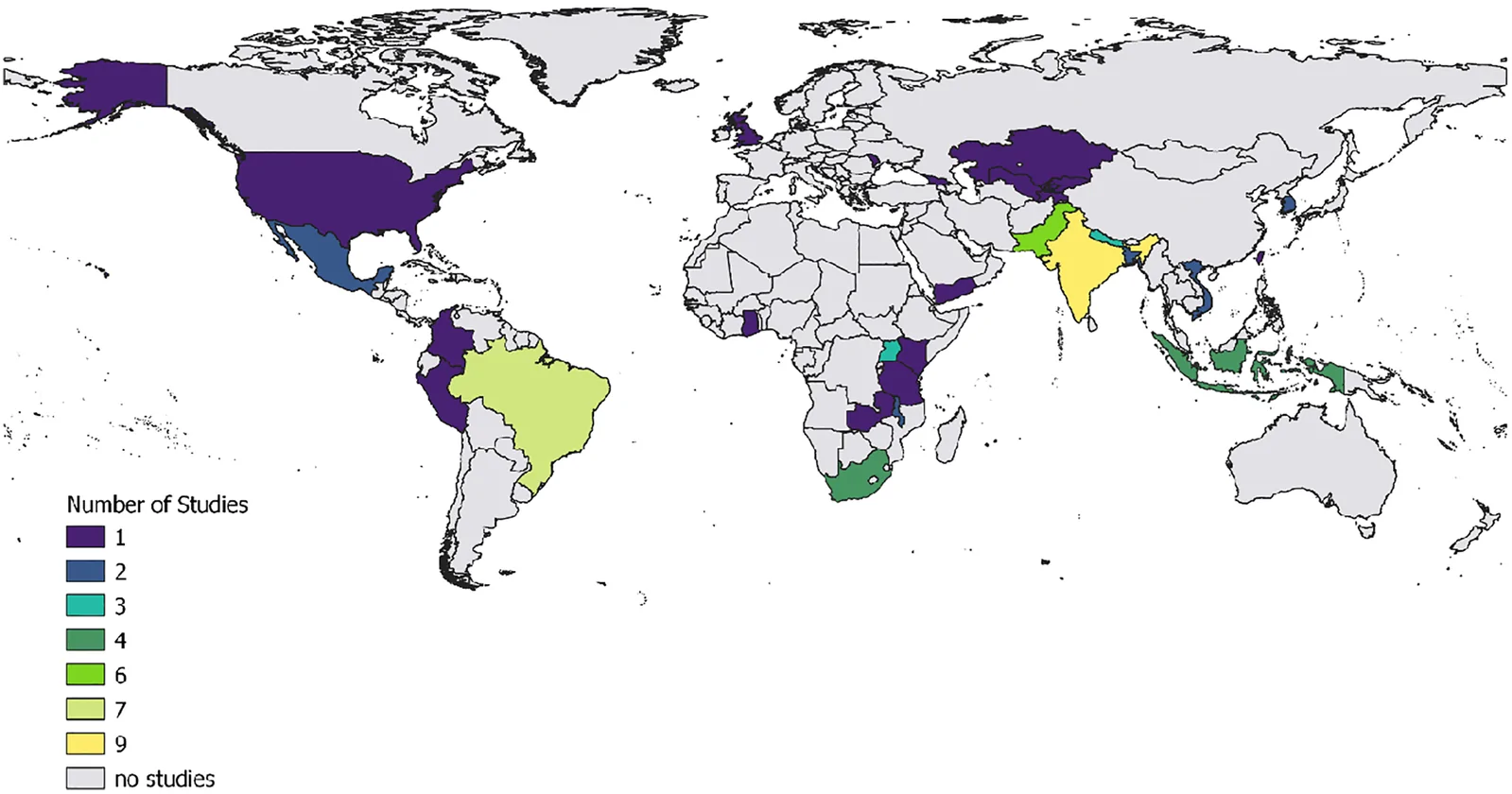
Global distribution of the included studies.

Out of 3204 papers following deduplication, 57 studies were included after full text screening. Results of the search strategy are detailed below (Fig 1). 38 studies focused on TB and 14 on leprosy. 26 studies presented data from both men and women (25 on TB and 1 on leprosy), 24 involved solely women (TB = 9, leprosy = 15) and 7 studies perspectives from men (TB = 6, leprosy = 1). Data stems from 29 different countries across 5 continents (Fig 2).

Around 1591 men and women participated in the included studies. Their ages ranged from 14-88 years old, most fell in the age range of 38–42 years. Participants included those who were newly diagnosed, currently in treatment, those who finished treatment, were lost-to-follow-up or relapsed. The majority of participants were married, with no formal education, and unemployed living in near poverty conditions or poverty. Most participants followed Islam, with less practicing Christianity and Hinduism. Not all studies reported demographic data (region, gender and age were reported in all studies, education was reported in 63% studies, employment in 68%, SES in 71%, religion in 49% and marriage in 59% of included studies.).

Countries are shaded according to the number of studies included from each country. Country boundaries are based on the Natural Earth Admin 0- Countries (1:10m) dataset (https://www.naturalearthdata.com/downloads/10m-cultural-vectors/10m-admin-0-countries/). Natural Earth data are in the public domain; terms of use: https://www.naturalearthdata.com/about/terms-of-use/.

### Quality appraisal

Overall, the methodological quality of the included studies was judged to be good, with the majority of studies meeting most of the CASP criteria (see S4 File). All studies clearly stated their aims and employed appropriate qualitative methodologies and research designs. However, a few recurring limitations were identified. Consideration of the researcher-participant relationship was often not reported (29/57 studies) and limitations or potential biases were not consistently addressed (21/57 studies). Additionally, recruitment strategies (7/5/), data analysis (11/57 studies) and ethical considerations (8/57) were occasionally inadequately described.

### Analysis of lived experiences

Narrative results are structured into 7 main themes (see Tables 2 and 3) showcasing the gendered experiences of people living with TB or leprosy: 1) Quality of Care 2) Health Behavior 3) Livelihood Effects 4) Psychological Effects 5) Effects on Relationships, 6) Coping and 7) Feedback and Recommendations (see S3 File, Synthesis of Study Results). Quotations are presented as illustrative examples to support the themes.

**Table 2:** Key gender differences in the lived experiences of TB and leprosy.

Theme	Women	Men
Quality of Care	More negative healthcare interactions, lack of gender-segregated TB treatment spaces, misdiagnoses more commonly experienced, specific for TB: classified as „disease of men “	Long waiting hours resulted in lost wages and work opportunities
Health-Behavior	Limited autonomy (permission, accompaniment, financial dependence), lower prioritization of own health, care-seeking motivated by family responsibilities	Care-seeking perceived as threatening masculinity, treatment framed as restoring masculinity, TB specific: adherence challenges linked to substance use
Livelihood Effects	Continued domestic responsibilities, illness concealed from husbands due to fear of divorce, Leprosy specific: concealment of appearance	Compelled to continue working, fear of job loss
Psychological Effects	Greater fear of rejection, guilt and shame, questioning femininity and motherhood, Leprosy specific: distress over disability and bodily changes	Distress linked to perceived loss of masculinity and economic consequences
Effects on Relationships	Higher stigma and family exclusion, severe marital consequences	Social consequences primarily linked to employment
Coping	Smaller and more fragile support networks	Broader support networks than women
Recommendations	Government-funded food and nutrition support linked to medication use, community- and family-centered education, outreach services to reduce childcare and transport barriers	Male-focused community outreach in social spaces (e.g., taverns, bars), peer support for clinic visits and sputum collection, workplace protections against mistreatment.

**Table 3:** Key differences and similarities in the lived experiences of TB and leprosy.

Key Differences		
**Theme**	**TB**	**Leprosy**
Quality of Care	Strong focus on health system barriers (crowding, privacy issues, no gender-separated treatment rooms, inconsistent HCW behavior, trust issues)	Less data on care experiences on system-level
Health Behavior	Highly gendered decision-making (husband permission, masculinity, work identity, competing work roles)	Less data on gendered decision-making dynamics, More self-treatment (herbs, creams)
Livelihood Effects	Strong work disruption and withdrawal from social life due to stigma	More adaptation/continued participation (especially men), emphasis on appearance and disability
Concealment and Disclosure	Driven by disease association stigma (e.g., fear of HIV link, employment loss, marital breakdown)	Driven mainly by visibility/body image (clothing, hiding physical signs)
Psychological Effects	Emphasis on fear, helplessness, and illness progression anxiety, masculinity questioned	Strong identity disruption (femininity, motherhood, body image)
Effects on Relationships	Marriage prospects affected, forbidden to marry someone with TB, behavior accusations	Preferred for WAL to also marry someone with leprosy
Coping	Peer-to-peer setting proved helpful	Emphasis on religion, acceptance, will-power, motherhood
Feedback and Recommendations	Clear recommendations (home outreach, outreach in common places, community interventions, targeted awareness, employment laws, village funds, food incentives, peer-to-peer support)	No recommendations reported
**Shared Findings**		
Quality of care	Stigmatizing or poor HCW behavior, medicine shortages, lack of information about side effects, limited trust, isolation, long waiting times, discomfort with disclosure, some positive compassionate care experiences	
Health Behavior	Delayed care-seeking (months–years), use of traditional healers, spiritual leaders, private providers, and self-medication before biomedical care	
Livelihood effects	Major difficulty in performing daily tasks and maintaining work and social roles	
Concealment	Widespread concealment of diagnosis, disclosure mostly limited to inner circle, fear of social rejection and relationship breakdown (especially divorce for women)	
Psychological Effects	High emotional burden: anxiety, sadness, distress, depression, guilt, self-blame, fear of death, infecting others, financial insecurity, suicidal thoughts, isolation	
Effects on relationships	Social rejection, stigma from peers, family strain, exclusion, marital disruption and gendered stigma patterns	
Coping	Religion, acceptance, self-care, maintaining routines, and reliance on social networks, psychosocial coping is central	

### Quality of care

A delay of diagnosis, ranging from months to years, was reported from both men and women affected by leprosy or TB [27–32], along with encountering wrong diagnoses along the way [29,32–35]. These challenges were described consistently across both diseases, indicating shared structural barriers to timely diagnosis. However, some gendered patterns were more prominently reported in TB studies. For example, women from eastern Europe, central Asia, India (Kolkata) and Brazil (Sao Paulo) described TB to be considered as a “disease of men”, thus leading to misdiagnoses for women more frequently [32–34]. Unsatisfactory interactions with the healthcare system upon diagnosis also appeared to be gendered. Across studies and regions, women affected by TB (WATB) were more frequently described rude behavior, scaremongering and disrespect by healthcare workers (HCW) when compared to men affected by TB (MATB) [34,36–40]. In contrast, evidence on such explicitly gendered negative interactions was less prominent in the leprosy literature.

Although studies on both diseases highlighted that men and women from all continents did not feel sufficiently informed by HCW about possible reactions or side effects from medication, which led to limited transparency and trust [28,29,32,34,36,38–44]. With that, three studies displayed that both genders felt uncomfortable with the fact that HCW disclosed their diagnosis to people in their community [29,45,46].

Structural impediments to high quality care were also reported across both diseases, although their manifestations varied. While WATB from Indonesia (Batanghari), India (Kolkata) and Peru (Ventanilla) pointed out a lack of privacy at crowded hospitals [34,38,47], MATB from England (London) and women affected by leprosy (WAL) from South Korea (Sorok Island) described extreme isolation in their hospital setting [44,48]. Additionally, WATB from Eastern Europe, Central Asia, and Yemen highlighted that treatment rooms were not separated for men and women, and that HCW were often male, which made them feel embarrassed [33,49]. Economic constraints linked to care-seeking were reported for both diseases, but were particularly emphasized among men. Men affected by leprosy (MAL) and TB in India (Assam), Malawi (Blantyre) and South Africa (Buffalo City) described long waiting times in outpatient departments (OPD), which resulted in a “lost day for work” [27,37,50]. Chronic shortages of medication were also reported across both diseases, further hindering access to consistent care [27,37].

Despite these challenges, some studies reported positive care experiences in both disease contexts. A study conducted in India (Assam) displayed positive care experience of both men and women affected by leprosy. HCW were described to be compassionate, taking sufficient time to explain procedures to patients. Women were said to be equally treated as men and sometimes even favored in terms of waiting times [27]. Guspianto et al. (2024) [47] and Onifade et al. (2010) [38] reported similar positive experiences for TB treatment in Indonesia (Jambi) and Peru (Ventanilla) [38,47] and HCW interactions were described as friendly, informative and supporting in TB studies across Asia, Africa and South America for both genders [28,30,32,36,40–42,47].

### Health behavior

#### Gendered decision-making power and care-seeking.

For TB, the majority of women explained that they needed to obtain permission from their husbands to seek care, and were often denied such [33,38,41,43,47,49,51–53]. MATB on the other hand were in control of their own treatment processes and mostly encouraged by their wives [41,50]. *“In my family, I am the boss, I decide […].” (*[41]*, p.484, MATB, Vietnam).*

In the included studies, a strong role of the women’s mother-in-law or other family members in women’s access to care was highlighted [33,36,38,54,55]. Similar dynamics were reported in the leprosy literature; however evidence was more limited, with only one study from India (Assam) describing comparable experiences among WAL [27].

Both WAL and WATB only sought care when symptoms started to worsen [28,30,32,40,48,55,56], similar to MATB [30,37,44,51,57]. WATB felt obliged to seek care in order to stay healthy for their families and children [38,47,51,55,58]. In contrast, MATB decided to seek care out of being unable to work or of having diminished marriage prospects [30,50].

Reasons not to seek care included family obligations for women across both diseases [28,33,34,38,48,55] and conflicting work obligations for MATB [30,33,37,38,50,51,59]. For TB, indirect financial burdens and opportunity costs, such as costs for treatment, transportation, missing work [27,28,32–34,37,38,40,41,46,51,54,55,59–61], as well as fear of being stigmatized were identified as barriers to care-seeking among both genders [33,37,39,46,50,54,55,57,62]. For men specifically, a few MATB from central Uganda and South Africa felt their masculinity threatened by illness and avoided the status of patient: *“[…] These men who are like girls are the ones who run to the hospitals.” (*[51]*, p.7, MATB, Uganda).*

For both diseases, women sought care later, either because of lower prioritization of their own health compared to other obligations, or because they required somebody to accompany them to the clinic [33,34,38,40,47–49,53–55,58,62,63]. Among those delaying seeking biomedical care, men and women reported that they consulted with traditional healers, private providers, spiritual leaders, or decided to self-medicate [28–30,33,34,37,42,54,57,59]. Specifically, for leprosy, women used creams, ointments, cement or ashes to treat skin rashes, or inhaled smoke from burning herbs “to expel toxins from their bodies” in Brazil (Cuiaba) and Taiwan (Lo-Sheng) [28,29].

#### Treatment adherence.

WATB and WAL stated that their adherence to treatment was motivated by a determination and desire to get cured, along with wanting to follow HCWs instructions accordingly [30,40,43,64,65]. However, beyond these accounts, evidence on treatment adherence among persons affected by leprosy (PAL) was limited in the included studies. In contrast, the TB literature provided more extensive insights. Both men and women affected by TB emphasized the importance of family support during treatment [43,50,66]. MATB were especially motivated to successfully complete treatment because they wanted to maintain their masculine identity and their role as fathers, as well as return to work [32,43,50].

Similar to care-seeking behavior, treatment adherence was affected by economic burden for men and women affected by TB [41,46,47,50–52,55,59,63,67]. Hereby, women were often dependent on their husbands’ income [47,55]. Furthermore, medication intake proved difficult for both men and women, as it often required them to avoid taking it on an empty stomach [34,50,59,68]. Single men from Brazil (Sao Paulo) were more likely to forget to take their daily medication, while men from India (Kerala and Bombay) reported interference with their alcohol or drug addiction [58,67]. WATB in Uzbekistan (Karakalpakstan) struggled with adherence due to family responsibilities. *“[…] I took the drugs for about 6 months and then left, being a defaulter…What they [family] say is: we need someone who will serve us.” (*[52]*, p.8, WATB, Uzbekistan).*Along with that limited family support, stigma and side effects posed as barrier to complete treatment among WATB [34,47,54,55,59,63,67].

### Livelihood effects

#### Adjustment to daily life during illness.

While one study reported that men affected by leprosy (MAL) from India (Assam) stopped doing household chores due to illness [27], WAL were generally expected to continue with their household responsibilities as usual, even when they were in pain [27,29,48]. A similar pattern was observed among WATB [34,43,44,47,55,67], who frequently reported continuing domestic work despite illness and functional limitations. Across both WATB and WAL, participants commonly expressed hardship in handling daily tasks due to impacted mobility and physical impairment after falling ill [28,34,43,47,48,55,65–67,69,70].

Differences were also observed in social participation. MAL from Nepal (Morang District) reported to still attend social activities, contrary to WAL who tried to avoid social life [27,29,45,71–74]. For TB, both men and women preferred to not attend social activities but rather keep to themselves to avoid discrimination [31,39–41,43,46,47,57,62,63,75,76].

In addition, severe functional consequences became evident among MATB. Unmarried men in South Africa (Gauteng and Eastern Cape) and Tanzania (Dar es Salaam) struggled with starvation on a daily basis, as the disease left them unable to cook and they had no one to cook for them [57,69,75].

#### Work & finances.

All participants, regardless of gender, and disease, faced loss of income and employment due to rejection, voluntary resignation to avoid stigma, frequent leave of absences, disability or the employers’ fear of infecting others [33,35,36,43,46,50,51,59,63,67,70,77,78]. *“[…] In the process of acknowledging his illness, he was fired to prevent other employees from getting TB and lowering work performances.” (*[75]*, p.3, MATB, South Africa)*. Next to that, some WATB, but mostly MATB were expected to keep working for the family [33,43,51,69]. Many men felt that they had no choice but to continue providing for their family because they were the family’s financial pillars [29,33,46,50,52,66,69]. *“Men think they are strong, the woman is waiting for you to bring money, you don’t have time to think, you have to work”. (*[33]*, p.6, MATB, Moldova).* For leprosy specifically, WAL from Brazil (Sao) and Taiwan (Lo-Sheng) explained that they had changed their type of employment to be more compatible with their symptoms and disabilities resulting from the disease [29,74].

#### Concealment and disclosure.

For both TB and leprosy, the majority of women and men decided to conceal their disease by isolating themselves from society [31,35,36,40,42–44,46,48,55,58,59,64,72,73,75,79–81]. Only a few disclosed their diagnosis to their inner circle of family and friends [57,58,72,75,76,78,79,81,82], though especially women decided not to tell their husbands fearing divorce [39,45,46,53–55,72,79]. For TB specifically, WATB concealed their disease to escape social consequences as a woman, like diminished marriage prospects, questioned ability to care for their husband and family, discrimination, judgement and exclusion [33,36,38,39,47,54,60–62,67,73,83], while men were concerned about losing employment after disclosure [41,43,44,57,58]. Furthermore, women and men affected by TB hid their disease because they were afraid of people linking it to human immunodeficiency virus (HIV) [44,61]. Disclosing their disease to others was not a concern for MATB in two studies [38,62]. Women with leprosy shared that they concealed signs of their disease by wearing long clothes and closed shoes [43,77,79], as well as telling those around them that they had a less stigmatized condition, like skin cancer or allergies [29,65,79].

### Psychological effects

#### Mental health.

Just under half of the studies reported that both men and women experienced sadness, loneliness, frustration, distress, anxiety and even depression due to the effects TB or leprosy had on their lives [27,28,30–32,36,39–42,44,45,47,48,64–66,72,76,79,84].*“[…] Even though [we are] physically free of the microorganisms, [we] would never be free of the leprosy emotionally.” (*[29]*, p.109, WAL, Taiwan).* Both genders expressed suicidal thoughts or made attempts at self-harm, often not going through with it because they did not want to burden their family further [27,29,31,36,39,40,45,47,48,66,72,76]. *“[…] I don’t want to live anymore, I want to commit suicide. Tomorrow, either I will leave this hospital, or my body will be taken out.” (*[36]*, p.8, WATB, Kyrgyzstan).* Additionally, WATB described feelings of helplessness [40,84] and fear, including fear of disease progression [39,40,76,77], while WAL indicated significant anger about being affected [48,64,72].

Across TB and leprosy, both genders feared death, infecting family and others, financial stability and rejection from their network [29,32,33,35,36,38,39,41,42,45,47,54,62,66,67,72,75]. However, only four studies reported on men worrying about rejection and stigma [42,75,78,82], whereas women expressed their fear of rejection, especially from their husbands, in one third of the studies [29,32,33,35,36,38,39,41,42,45,47,54,62,66,67,72,75]. Specifically, for leprosy, women voiced concerns about disabilities and physical changes [28,29,45,48], whereas WATB worried about their inability to care for their family, being dismissed from work, and people associating their TB diagnosis with HIV [42,46,55,58,61,66,67,83,84].

#### (Distorted) self-image.

Numerous studies reported that WAL and WATB frequently internalized stigma and blame, which manifested as feelings of guilt, shame, embarrassment, and insecurity [32,35,36,39,40,42,45,48,55,64,65,68,72,73,75,79]. While this was also observed in three studies of MATB, it was more commonly found among women [33,59,75]. For men, the tendency was to suffer from a lack of masculinity due to perceived weakness and inability to provide [31,50,59,62,75,82]. Similarly, WAL questioned their femininity and role as mothers. They felt that they were not as competent as other mothers, wives or women in general because of the disease [29,35]. *“I do not find myself a complete woman because of the disease. “[…] A real woman is a healthy woman, I differ from others […]” (*[77]*, p.382, WAL, Brazil).* Complemented by that, changes in appearance resulted in an impaired self-image for women, especially for those affected by leprosy [32,33,39,42,49,52,69,74,77–79,81]. For instance, WAL used terms like “rotten”, “frog leather”, “snake skin” and “rotten bean” to describe their looks [77]. Effects on appearance due to excessive weight loss were also reported among WATB, although less common [38,75].

### Effects on relationships

#### Community.

The majority of women affected by TB or leprosy faced social stigma, including verbal abuse, rejection and judgement from colleagues, friends and neighbors [29,32,35,36,38–40,43,45–48,55,59,61,64–67,72,73,75,77,80,81,84]. *“If they know you have TB they start treating you like you’re not a human being like them.” (*[44]*, p.144, WATB, Uganda).* For men, on the other hand, studies were evenly divided between those reporting that men encountered social stigma [31,44,46,75,82,83], and those concluding that they did not [27,38,59,69,78,84], especially for MATB. However, in one study men were incorrectly accused of contracting TB based on their drinking, smoking and partying habits [48]. *“The social effects sometimes are worse than the illness itself […].” (*[49]*, p.5, MATB, Yemen)*

#### Marriage and partnerships.

More women [29,38–40,43,66–70,72,76,79,81,83] than men [50,60,75,78,82] shared that being affected by TB or leprosy had a negative impact on their marriage. Across both diseases, women narrated that their husbands became distant and avoidant, some experienced domestic violence or sexual abuse [69,79,81,83]. For women, being affected by TB or leprosy often resulted in divorce [33,36,41,43,46,53,54,61,62,66–68,76,78,83] or their husbands taking a second wife [43,60,61,68,81]. *“When my husband heard about the disease, he immediately sent me back home, saying he did not want a sick and useless wife with doubtful loyalty.” (*[54]*, p.10, WATB, India).* Three studies involving men showed that TB or leprosy had no impact on their marriages [67,78,84], along with two studies involving women [79,83].

Nevertheless, these patterns were reported across both TB and leprosy studies, however, some differences in contextual emphasis were observed. For TB, a study from Vietnam (Quang Ninh Province) found that ending a marriage by using TB as the reason was only an option if the affected person was a woman [41]. Two studies displayed that having children was a factor in preventing husbands from leaving [67,83]. In contrast, MATB attributed the dissolution of their relationship to the fact that the disease had impacted their sexual performance [57,75,82]. Furthermore, the marriage prospects of women were affected by the diseases, especially WATB voiced concerns [33,35,39,46,54,60,62,68,69].*“[…] Who will marry a diseased girl?” (*[62]*, p.1112, WATB, Bangladesh)*. Only one study found that men voiced concerns about their ability to get married because of being affected by TB [69]. Women on the other hand shared that their marriage proposal had been called off due to being affected by TB [39,54,66]. *“[…] If a boy had TB he may still get married, but if a girl had TB the boy’s family would not want to fix marriage with her.” (*[39]*, p.731, WATB, India).*

Four studies with WATB explained that it was prohibited to marry into a family affected by TB [59,60,66,69]. Contrary to that, one study involving WAL showed that families preferred their daughters to marry men with a history of leprosy [72].

#### Family and in-laws.

Both men [46,50,54,75,82] and women [27–29,35,38–40,43,54,60,63,66,68,73,80,84] faced avoidance, hostility, exclusion and isolation from their families across diseases, however, especially WATB [27,28,38–40,43,54,60,63,66,80,84].*“[…] [I] had been served food in a dog’s dish.” (*[68]*, p.122, WAL, India).* In addition, women experienced neglect and mistreatment at the hands of their in-laws [27,34,36,81], particularly their mother-in-law, who discriminated against them, spoke to them in demeaning ways and restricted their autonomy and participation in daily activities [34,36,39,67,83]. Three studies with MATB [32,41,80], three studies with WATB [36,61,83] and one study with WAL [29] reported positive family attitudes towards the participants and their disease.

### Coping

Mostly women shared a number of coping strategies that helped them overcome the hardships faced by the disease. For instance, religion and believing in a higher power proved helpful, particularly for WAL [29,35,36,39,45,48,65,73,77], along with inner acceptance and willpower [39,40,45,65,77]. WAL also mentioned their social role as a mother as an important coping mechanism, leaving them no choice but to “carry on with things” [73,74]. Furthermore, self-care, listening to music, watching TV, reading books and maintaining responsibilities helped distract WAL and WATB from their situation [36,39,77]. Specifically for TB, men and women described how sharing their experiences with others in a peer-to-peer setting brought them joy and relief [36,39,82]. Finally, it was identified that support from family, spouses, communities and HCW was a key coping mechanism for both genders across diseases. This included financial and emotional encouragement, helping with work, accompanying them to treatment along with caring and cooking for children. For both leprosy and TB, numerous studies found that both men and women received such support [27,29,30,32,36,39,43,45,46,49,50,52,55,57,64,67,70,75,78–80,83], however women’s support networks were often smaller than men’s [32,38–40,43,47,49,51,67].

### Feedback and recommendations

Participants shared a few recommendations that they felt would benefit them and others that are living with the disease. Suggestions were only provided by those affected by TB. People affected from Malawi (Blantyre), Peru (Ventanilla) and the Philippines (Paytas) recommended outreach services, whereby HCW would visit people at home for treatment, to ease difficulties with childcare work, and transportation [37,38,55]. Furthermore, participants from two studies in Malawi (Blantyre) and South Africa (Eastern Cape) suggested large-scale community and family-centered awareness-raising and educational interventions to foster gradual reforms [37,82]. In Malawi, men and women urged the government to introduce employment laws that would penalize supervisors for mistreating employees, enroll everyone in a medical scheme, refund transport costs to encourage care seeking, and establish village funds, where everyone contributes a small amount to pay for transport and ambulances [37]. Women from India (Kolkata), also asked for improved nutrition for women through government-funded food incentives, as this is important for medication intake [34]. Furthermore, participants in Malawi disliked the fact that researchers portrayed traditional medicine as wrong and as something that people should be punished for [37]. Women and men from South Africa (Eastern Cape) and Zambia (Lusaka) recommended spreading information to ensure early diagnosis in common social and comfort spots. For men, this would be in taverns and bars, where cigarettes and beers are shared. Women on the other hand could be reached at church [30,82]. Finally, men from South Africa expressed their desire and willingness to support other affected men by accompanying them to the clinic and helping with sputum collection [82].

## Discussion

This systematic review set out to examine how gender shapes the lived experiences of persons affected by TB or leprosy across diverse geographic and sociocultural contexts. While both genders experienced delayed diagnosis, limited trust in HCW, economic constraints, concealment issues, strained relationships, stigma, disruptions to daily livelihoods and psychosocial distress, women were found to be more disadvantaged than men across all themes. Particularly, in regard to stigma, mental health, access to care, social support and autonomy (Table 2). These patterns were observed across both TB and leprosy, as both diseases share a number of similarities due to the shared socio-economic circumstances associated with PRD. At the same time, a few differences emerged (Table 3). It is important to consider that more TB-related studies were included in the review, therefore, evidence was not equally divided between the two diseases. With this in mind, persons affected by TB (PATB) highlighted gendered decision-making power in care-seeking and the impact of stigma on employment and marital prospects more consistently than PAL. Furthermore, PATB more commonly engaged in social withdrawal and were afraid to disclose their disease, fearing that it would be linked to HIV. In contrast, concealment for leprosy placed a stronger emphasis on hiding visible bodily changes. Appearance played a particularly important role for PAL, impacting their identity, specifically in relation to femininity and motherhood. Coping strategies, like religion and acceptance were predominantly mentioned in relation to leprosy, but less so in relation to TB.

### Gender and health

It became evident that health disparities are not merely disease or context specific, but deeply embedded in gendered power relations and normative expectations [85]. This aligns with the broader literature on gender and health, which highlights how gender norms influence health behaviors, opportunities and outcomes [86]. With men in many contexts having to demonstrate “real masculinity” by not showing any weakness in times of sickness, women are expected to carry on with their feminine roles by prioritizing their family’s needs over their own health [20,87]. Deviating from such norms due to illness may have serious physical, and psychosocial consequences for those affected, as demonstrated in this study and supported by numerous others in the global literature [20,86,88,89]. Given that power, status and resources are distributed differently within the gendered social system, in which masculine traits are considered superior to feminine ones, women face greater vulnerability [20]. These vulnerabilities are for example reflected in healthcare interactions: where women’s physical symptoms are often dismissed as psychosomatic, due to stereotypes of emotional fragility [20]. This is also represented in this review, given the higher rate of misdiagnosis among women. Conversely, men’s presentations are often taken more seriously as they are characterized as strong and unlikely to seek care unless severely ill [20, 88]. Moreover, women are disproportionately more affected than men by mental health conditions worldwide, with disability adjusted life years (DALY) rates being one third higher among women than men [90]. However, these vast differences may be partly influenced by gender-related reporting biases, as men are often reluctant to disclose mental distress due to social norms that emphasize strength and self-reliance [91]. In line with other studies, our findings further showed that women were more likely than men to worry about social rejection and stigma during and beyond times of illness, which may contribute to their higher observed mental health burden [19,36,92,93].

### Intersectionality and gender

Such disparities are amplified when gender intersects with other social determinants of health (SDH), such as socio-economic status, sexuality, age, ethnicity, and disability, further shaping access, treatment and health outcomes rather than acting as an isolated determinant [94]. As TB and leprosy are both classified as PRD, these intersecting dynamics are particularly pronounced, as structural poverty, social marginalization, and constrained health systems resources overlap with, and amplify gendered inequalities [6]. This is also reflected in the demographics of the study population, with most of the participants belonging to a lower SES.

Within this intersectional context, our findings suggest that gendered social roles interact closely with economic insecurity in shaping health behavior, which emerges as particularly influential. Confirming our results, multiple studies show that men in poor socio-economic circumstances often feel they have no choice but to keep working instead of seeking care, in order to fulfil their role as husbands and fathers who can provide for their families, thus delaying care or abstaining from treatment [19,50,88,95]. On the contrary, these same gendered economic structures constrain women’s access to care in different ways. In line with our findings, treatment and transport costs have repeatedly been found to be a key barrier to care for women in 65 LMICs [96]. With a majority of women being financially dependent on men due to their social role, their autonomy over health decisions is ultimately restricted. However, studies from sub-Saharan Africa and Pakistan have shown that improving women’s access to financial resources and decision-making power positively impacts their use of healthcare [97,98]. This aligns with a broader body of evidence, indicating that women are generally more likely than men to engage in health-promoting behaviors, however, gendered social roles continue to prevent them from achieving equitable or better health outcomes [99]. Women therefore experience overlapping and intersecting forms of disadvantage in relation to disease, a phenomenon described in the literature as “triple jeopardy”. A number of studies suggest that WATB and WAL more commonly experience rejection and stigma from their social networks [15], negatively impacting their self-esteem, social participation and marital prospects [15,68,100–102]. In addition, evidence indicates that women are more likely to experience delays in care-seeking and diagnosis, and show greater tendencies to conceal their illness [15,102–104]. This aligns with broader evidence showing that while women generally have a higher life expectancy, they spend more years in ill health and disability [90]. The past 30 years have shown that global health progress has not been even between genders [90]. Understanding the diverse health needs and the different ways in which disease is experienced across gender is therefore essential in order to recognize key health opportunities.

### TB and leprosy: Similarities and differences

While many findings converged across TB and leprosy, a few important differences emerged, particularly in relation to visibility of symptoms, gendered social consequences and social withdrawal. Although gender acts as a dominant factor in shaping the lived experiences and health outcomes of those affected by TB or leprosy, it does not operate in isolation from disease-specific characteristics. In addition to the well-established similarities in the etiology of the two diseases [9], this study revealed that persons affected by either TB or leprosy commonly face similar socio-economic and gender-related challenges. Although the bodily effects of leprosy are amplified, especially for women, who feel that their feminine appearance has been taken away, the impact on appearance due to weight loss also played a part for participants affected by TB. Interestingly, according to the evidence presented in this review, both diseases appear to be associated with comparable levels of internalized and social stigma, although underlying drivers and expressions may potentially differ. For TB, stigma is often associated with high contagiousness of the disease and its link to HIV, while for leprosy, physical deformity affects stigmatizing beliefs. This is despite the fact that leprosy is classified as low-endemic in a majority of countries and is considered neglected [4], whereas TB is described as one of the most well-known diseases worldwide, belonging to the top three leading causes of death globally [2]. Consequently, it can be assumed that in this case, stigma is not entirely proportional to disease prevalence or visibility, nor is it fully defeated by biomedical or epidemiological progress/success. Instead, it appears to be predominantly rooted in social determinants, and the social and cultural meanings attached to the two PRD. Furthermore, the differing contagiousness of the two diseases, with TB being far more contagious, did not appear to substantially alter participants‘ reported experiences of stigma in this review, however the commonly assumed link between TB and HIV strongly affected social participation for PATB. Additionally, participants reported delayed- and misdiagnosis equally across diseases, although the literature suggests that delays are longer for PAL than PATB, taking months to years compared to weeks to months [105,106].

Given the long history of leprosy and TB and their co-evolvement with humanity over thousands of years [107,108] as well as the socio-economic similarities in the lives of those affected by them, it stands to reason that global efforts to combat these diseases may benefit from learning from each other. Combined TB and leprosy programs are already in place in a handful of African and Asian countries, including Tanzania, Uganda, Nigeria and Bangladesh [109–112]. These programs benefit from shared infrastructure, diagnostic overlap and improved service delivery, as integrating leprosy care into general health facilities becomes easier when linked to TB. Cost-effectiveness and active case finding are also improved, especially for PAL, since TB screening is more common due to its high prevalence and can result in the detection of leprosy cases, which are comparatively rarer [113–115]. Altogether, these findings suggest that while disease-specific characteristics shape certain aspects of lived experience, the overarching patterns are more strongly driven by shared socio-economic conditions and gendered social structures. In this sense, the classification of both diseases as PRD provides a useful lens to understand why similarities in lived experiences outweigh disease-specific differences in this review. However, these similarities should not obscure important distinctions between the two diseases, particularly regarding disability-related stigma in leprosy and gendered perceptions of TB as a “male disease”, which may influence diagnosis and care pathway differently.

### Recommendations for policy and practice

Based on the points discussed and the feedback on care provided by participants under theme 7, the following recommendations can be derived for policy and practice (see Box 1).

**Box 1. Key Recommendations for Policy and Practice**

**Recommendations for Policy and Practice to Improve Lived Experiences for People Affected by TB or Leprosy**

- **Adopt gender-sensitive and intersectional approaches** recognizing the interaction between gender, socioeconomic status, geography, and health system access in shaping health experiences.
- **Reduce indirect costs of care by addressing transportation expenses**, income loss, and caregiving responsibilities through financial protection and support mechanisms.
- **Strengthen outreach and decentralized care**, including home-based services and mobile clinics, particularly in rural and underserved settings.
- **Improve service accessibility and acceptability** through flexible clinic hours, reduced waiting times, and safe, gender-responsive care environments.
- **Integrate TB and leprosy services where appropriate**, ensuring context-specific implementation that reflects epidemiological and health system differences.
- **Enhance health care worker training** on stigma, mental health, and gender-specific barriers to improve communication, trust, and early diagnosis.
- **Promote early diagnosis and community awareness** through targeted outreach in everyday social spaces and tailored communication strategies.
- **Strengthen psychosocial and peer support systems**, including self-help groups and community-based mental health support in resource-limited settings.
- **Empower affected individuals**, particularly women, through financial support, education, vocational training, and involvement in program design.
- **Engage communities** through participatory approaches, including involvement of affected persons in stigma reduction and health promotion activities.

Recognizing TB and leprosy beyond their physical impact and suggested treatment regimen remains essential to improving the lives of those affected. This is especially important for PRD like TB and leprosy, whose epidemiology cannot be separated from the SDH that amplify or mitigate them. Although direct costs are widely covered for Multi-Drug-Treatment (MDT), the indirect costs to those affected need to be acknowledged further. This includes transport costs, loss of income, and caregiving responsibilities, which were consistently reflected in the findings. This could be achieved by offering more outreach services, particularly in rural areas, providing care outside of working hours, improving childcare and creating gender-responsive and accessible spaces. A recent study from Pakistan, for example, showed that HCW visiting PAL on Sundays improved detection and treatment rates [19].

In this regard, it is important to note that PRD are shaped by broader social and structural determinants, underscoring that care-seeking and treatment adherence should not be conceptualized solely as an individual responsibility. These structural barriers are further compounded by intersecting factors such as geographic remoteness, disability, and life-stage related vulnerabilities, which further shape access to care and treatment adherence [6,116].

Furthermore, as previously mentioned, combining TB and leprosy programs may improve cost-effectiveness due to shared infrastructure and awareness campaigns, as well as increased outreach and early case detection. A number of countries across Africa and Asia reported promising benefits of combined programs, as previously discussed [109–112]. Although TB programs receive substantially greater global financial investment than leprosy programs, evidence that TB financing has been directly used to support leprosy programs remains limited [117]. However, this should be implemented in a context-sensitive manner, as differences in epidemiology and health system capacity remain relevant despite similarities in lived experience. Moreover, especially for leprosy, integrating leprosy programs into general rehabilitation services and national NTDs programs, as implemented in Togo in 2018, may improve utilization of resources for the disease [118].

Moreover, the quality of care could be improved further by sensitizing and educating HCW to recognize mental health issues and internalized stigma related to the disease. Several studies reported that HCW-patient relationships improved after HCW received psychological training [119,120]. Along with that, disease self-management and trust in treatment procedures were reported to improve when HCW adapted more positive and empathetic attitudes toward their patients [79,121–124]. Next to HCW, the establishment of self-care groups, peer-to-peer-support, or training lay community members as mental health counsellors can close an important gap in settings where mental health resources are limited. This has proven effective in a number of LMIC [125,126].

Alongside this, misdiagnosis and delayed diagnosis could be reduced by creating more knowledge around gender specific disease characteristics, especially among women, who in many contexts experience dismissal of symptoms and delayed diagnostic pathways. A study from Nepal, for example, addressed this by training female community health volunteers to recognize signs of leprosy, which increased women’s comfort in seeking care [127].

Empowering girls and women by creating safe care spaces, providing financial and educational support programs, and promoting self-advocacy should play a key role going forward. Evidence showed that vocational training and creating self-help groups were associated with improved self-esteem and social participation among women [127]. Nevertheless, gender inequalities are deeply embedded in the sociocultural context of countries, and are perpetuated by wider policies, making it admittedly difficult to tackle. However, a study investigating successful gender integration across five UN agencies, found that action and change can be derived from raising more awareness through data and evidence collection, integrating civil society organizations into institutional structures, and harnessing the power of the collective action [128].

Furthermore, according to our results, a functional support system appeared to be the most relevant aspect of the coping process, therefore a primary focus should be placed on it. Employing a participatory approach by engaging all relevant stakeholders and recipients, especially those with in leading community roles, can facilitate trust, awareness, and greater understanding within social networks. A study from India, for example, facilitated meetings between PAL, community members, and influential “leaders”, where common misconceptions were addressed, which improved attitudes toward PAL and increased awareness in the community [129]. Furthermore, engaging with people affected by TB or leprosy may help to humanize aspects of the disease, combat stigma and misinformation, and foster a sense of belonging. In this regard, a recent study from South America, Africa and South Asia has shown that participatory storytelling can be effective in giving voice to people affected by NTDs, empowering them socially and fostering solidarity [130].

### Strengths and limitations

This systematic review has several strengths and limitations that are worth to be considered. The review presents a large and diverse body of evidence, including 57 studies from 29 countries across 5 continents. While the majority of gender-focused studies primarily focus on women, this review enabled comparisons to be made between men and women. However, other gender identities could not be investigated due to a lack of evidence in the literature. Furthermore, the lack of research on gender and leprosy, particularly among MAL, resulted in a greater inclusion of TB-related gender studies among women. It is therefore important to interpret findings in light of the fact that findings are not equally divided by disease. As only qualitative studies and the qualitative components of mixed-method studies were included, the results are subject to inherent bias and may not be as generalizable. Nevertheless, the overall quality of the studies was rated as good with relatively recent study data (see S4 File, Quality Appraisal of Included Studies). However, the CASP appraisal identified recurring limitations. In particular, many studies did not explicitly consider the researcher-participant relationship, and reporting of analytical procedures and study limitations was sometimes limited. In some cases, ethical considerations and recruitment strategies were also insufficiently described. These factors may affect transparency and depth of the original analyses. While no studies were excluded based on their quality, these limitations were taken into account when interpreting the findings and the presented results should be considered in light of them. Due to resource constraints, the final quality appraisal of the studies was conducted by one author only, which may introduce subjective bias. However, the use of the CASP checklist provided a structured and consistent framework for appraisal across all studies. Furthermore, it should be noted that SCWU was co-author on two included studies [36,66], however the author’s contribution to these studies was limited to administrative support, editing of the manuscript, and assistance in the development of the methodology. Moreover, primary coding and synthesis were conducted by the first author, due to resource constraints, which may limit interpretive breadth, however reflexive thematic analysis acknowledges the researcher as the primary analytic instrument, and rigor was supported through a transparent, stepwise analytical process and interpretive input from co-authors during different stages of analysis. The research team comprised women from Germany and Tanzania with complementary research and field experience in TB and leprosy. While none of the authors are personally affected by either disease and our cultural backgrounds differ from those of many study participants, we acknowledge that our positionality may have influenced interpretation. Regular reflexive discussions among the authors were therefore used to critically examine assumptions and consider alternative interpretations.

Despite these limitations, this review provides novel and valuable insights, stemming directly from the lived experiences of men and women affected by TB or leprosy.

## Conclusions

This systematic review highlighted that gender-based health disparities are pervasive and reinforced across diseases, populations and different regional contexts. While both men and women experienced delayed diagnosis, stigma, economic hardship, and psychosocial distress, women were disproportionately affected in almost all areas. These inequities are not solely attributable to disease pathology, but are deeply embedded in gendered social norms, power relations, and social and structural determinants of health. These dynamics operated across both TB and leprosy, although the degree and expression of disadvantage varied by disease context and setting.

Notably, despite the differences in epidemiology, chronicity, and visibility between TB and leprosy, findings showed substantial overlap in lived experiences, particularly regarding stigma, socio-economic disruption, and psychological distress. This suggests that especially stigma is not primarily driven by biomedical characteristics alone, but is socially produced and shaped by shared SDH, such as poverty, marginalization and gender inequality. At the same time, disease-specific differences remain important and should not be overlooked. By synthesizing qualitative evidence from two historically intertwined diseases, this review provides a gender-centered and comparative perspective that has largely been missing from the literature.

The findings suggest that TB and leprosy programs can learn from one another, particularly regarding stigma reduction, community engagement, integrated service delivery, and support mechanisms that address the indirect costs and social consequences of illness. At the same time, such integration should remain context-sensitive, acknowledging epidemiological and health system differences between the two diseases. Ultimately, tackling TB and leprosy requires moving beyond biomedical models and adapt interventions that explicitly recognize gender as a key SDH. Integrating gender-sensitive, participatory, and intersectional approaches into policy and practice is essential to reducing inequality, and improving early diagnosis, treatment adherence, and long-term outcomes for all people affected. Overall, this review underlines that TB and leprosy should be understood in relation to shared social and structural conditions, rather than being viewed only through their clinical features. This emphasizes how diseases that appear very different in terms of their global health impact can still be remarkably similar in their lived experiences shaped by these determinants.

## Supporting information

S1 PRISMA Checklist From: Page MJ, McKenzie JE, Bossuyt PM, Boutron I, Hoffmann TC, Mulrow CD, et al. The PRISMA 2020 statement: an updated guideline for reporting systematic reviews. BMJ 2021;372:n71. https://doi.org/10.1136/bmj.n71. This work is licensed under CC BY 4.0. To view a copy of this license, visit https://creativecommons.org/licenses/by/4.0/.
(DOCX)

S1 File Search Strategy. (DOCX)

S2 File Data Extraction. (DOCX)

S3 File Synthesis of Study Results. (DOCX)

S4 File Quality Appraisal of Included Studies. (DOCX)

S5 File Coding Framework. (DOCX)
