# Supplementary material for: Gendered experiences of people living with tuberculosis or leprosy: A global systematic review
Source: PLoS Negl Trop Dis. 2026 Sep 8;20(9):e0014711. doi: 10.1371/journal.pntd.0014711 (PMC13577493; doi:10.1371/journal.pntd.0014711)
Supplement: Supplementary file 2 [file pntd.0014711.s002.docx]

**S1 File**

**Search Strategy**

**1. PubMed**

| **Data Base** |  | **Search Strategy** | **Date** | **Results** |
| --- | --- | --- | --- | --- |
| Pubmed | **#1** | "leprosy"[MeSH Major Topic] OR "Hansen's disease"[Title/Abstract] OR "lepros*"[Title/Abstract] OR "tuberculosis"[MeSH Major Topic] OR "tuberculosis"[Title/Abstract] OR "TB"[Title/Abstract] | 4.08.2025 | 328,539 |
|  | **#2** | "sex factors"[MeSH Terms] OR "gender identity"[MeSH Terms] OR "Sex Characteristics"[MeSH Terms] OR "Sexual and Gender Minorities"[MeSH Terms] OR "Sexuality"[MeSH Terms] OR "female"[MeSH Terms] OR "male"[MeSH Terms] OR "gender"[Title/Abstract] OR "women"[Title/Abstract] OR "men"[Title/Abstract] OR "female"[Title/Abstract] OR "male"[Title/Abstract] OR "transgender*"[Title/Abstract] OR "transsexual*"[Title/Abstract] OR "intergender"[Title/Abstract] OR "intersex*"[Title/Abstract] OR "bisexual*"[Title/Abstract] OR "queer"[Title/Abstract] OR "homosexual*"[Title/Abstract] OR "lesbian*"[Title/Abstract] OR "non-binary"[Title/Abstract] OR "nonbinary"[Title/Abstract] OR "sex difference*"[Title/Abstract] OR "sex"[Title/Abstract] OR "sexual*"[Title/Abstract] |  | 14,284,128 |
|  | **#3** | "lived experience*"[Title/Abstract] OR "life experience*"[Title/Abstract] OR "patient experience*"[Title/Abstract] OR "personal experience*"[Title/Abstract] OR "illness experience*"[Title/Abstract] OR "experiences"[Title/Abstract] OR "experience"[Title/Abstract] OR "experiential"[Title/Abstract] OR "life-world"[Title/Abstract] OR "life-world"[Title/Abstract] OR "daily life"[Title/Abstract] OR "living with"[Title/Abstract] OR "stigma*"[Title/Abstract] OR "perceived"[Title/Abstract] OR "perception*"[Title/Abstract] OR "perspective*"[Title/Abstract] OR "understanding"[Title/Abstract] OR "journey"[Title/Abstract] OR "thematic"[Title/Abstract] OR "theme*"[Title/Abstract] OR "narrative*"[Title/Abstract] OR "narration"[Title/Abstract] OR "life stor*"[Title/Abstract] OR "psychosocial"[Title/Abstract] OR "emotional"[Title/Abstract] OR "identity"[Title/Abstract] OR "meaning"[Title/Abstract] |  | 3,992,352 |
|  | **#4** | "Qualitative Research"[MeSH Terms] OR "Interviews as Topic"[MeSH Terms] OR "Focus Groups"[MeSH Terms] OR "Narration"[MeSH Terms] OR "Ethnology"[MeSH Terms] OR "Grounded Theory"[MeSH Terms] OR "qualitative"[Title/Abstract] OR "interview*"[Title/Abstract] OR "focus group*"[Title/Abstract] OR "ethnograph*"[Title/Abstract] OR "Grounded Theory"[Title/Abstract] OR "narrative*"[Title/Abstract] OR "thematic analysis"[Title/Abstract] OR "content analysis"[Title/Abstract] OR "phenomenolog*"[Title/Abstract] OR "life history"[Title/Abstract] OR "semi-structured"[Title/Abstract] OR "in-depth"[Title/Abstract] OR "open-ended"[Title/Abstract] OR "case study"[Title/Abstract] OR "case studies"[Title/Abstract] OR "participant observation"[Title/Abstract] OR "autoethnograph*"[Title/Abstract] |  | 1,206,151 |
|  | **#5** | "journal article"[Publication Type] |  | 36,689,849 |
|  | **#6** | 1995/01/01:3000/12/31[Date - Publication] |  | 27,913,229 |
|  | **#7** | "english"[Language] OR "german"[Language] OR "french"[Language] OR "spanish"[Language] OR "portugese"[Language] |  | 36,296,394 |
|  | **#8** | "all"[Filter] NOT ("animals"[MeSH Terms] NOT "humans"[MeSH Terms]) |  | 33,806,995 |
|  | **SEARCH**  **Without**  **Qualitative**  **Block** | #1 AND #2 AND #3 AND #5 AND #6 AND #7 AND #8 |  | 7,256 |
|  | **SEARCH With Qualitative Block** | #1 AND #2 AND #3 AND #4 AND #5 AND #6 AND #7 AND #8 |  | 1,603 |
|  | **SEARCH without language restriction** | #1 AND #2 AND #3 AND #4 AND #5 AND #6 AND #8 |  | **1,648** |

**2. Web of Science**

| **Data Base** | **Search** | **Search Strategy** | **Date** | **Results** |
| --- | --- | --- | --- | --- |
| Web of Science | **#1** | TS=(leprosy OR "Hansen's disease" OR lepros* OR tuberculosis OR TB) | 4.08.2025 | 308,200 |
|  | **#2** | TS=(gender OR "gender identity" OR "sex characteristics" OR "sex factors" OR "sexual and gender minorities" OR sexuality OR women OR men OR female OR male OR transgender* OR transsexual* OR intergender OR intersex* OR bisexual* OR queer OR homosexual* OR lesbian* OR "non-binary" OR nonbinary OR "sex difference*" OR sexual*) |  | 6,100,890 |
|  | **#3** | TS=("lived experience*" OR "life experience*" OR "patient experience*" OR "personal experience*" OR "illness experience*" OR experiences OR experience OR experiential OR "life-world" OR "daily life" OR "living with" OR stigma* OR perceived OR perception* OR perspective* OR understanding OR journey OR thematic OR theme* OR narrative* OR narration OR "life stor*" OR psychosocial OR emotional OR identity OR meaning) |  | 15,028,180 |
|  | **#4** | TS=(qualitative OR interview* OR "focus group*" OR ethnograph* OR "grounded theory" OR "thematic analysis" OR "content analysis" OR phenomenolog* OR "life history" OR "semi-structured" OR "in-depth" OR "open-ended" OR "case study" OR "participant observation" OR autoethnograph*) |  | 2,916,768 |
|  |  | Filter: Refine by Documentation Type “Article”  Date Range: 1995-01-01 to 3000-01-01 |  |  |
|  | **Final Search** | #1 AND #2 AND #3 AND #4 |  | 1038 |

**3. Scopus**

| **Data Base** | **Search** | **Search Strategy** | **Date** | **Results** |
| --- | --- | --- | --- | --- |
| Scopus | **#1** | (TITLE-ABS-KEY(leprosy OR "Hansen's disease" OR lepros* OR tuberculosis OR TB)) | 4.08.2025 |  |
|  | **#2** | (TITLE-ABS-KEY(gender OR "gender identity" OR "sex characteristics" OR "sex factors" OR "sexual and gender minorities" OR sexuality OR women OR men OR female OR male OR transgender* OR transsexual* OR intergender OR intersex* OR bisexual* OR queer OR homosexual* OR lesbian* OR "non-binary" OR nonbinary OR "sex difference*" OR sexual*)) |  |  |
|  | **#3** | (TITLE-ABS-KEY("lived experience*" OR "life experience*" OR "patient experience*" OR "personal experience*" OR "illness experience*" OR experiences OR experience OR experiential OR "life-world" OR "daily life" OR "living with" OR stigma* OR perceived OR perception* OR perspective* OR understanding OR journey OR thematic OR theme* OR narrative* OR narration OR "life stor*" OR psychosocial OR emotional OR identity OR meaning)) |  |  |
|  | **#4** | (TITLE-ABS-KEY(qualitative OR interview* OR "focus group*" OR ethnograph* OR "grounded theory" OR "thematic analysis" OR "content analysis" OR phenomenolog* OR "life history" OR "semi-structured" OR "in-depth" OR "open-ended" OR "case study" OR "participant observation" OR autoethnograph*)) |  |  |
|  |  | Date range: 1995-2025, Document Type: Article |  |  |
|  | **Final Search** | #1 AND #2 AND #3 AND #4 |  | 2370 |

**4. Embase**

| **Data Base** | **Search** |  | **Date** | **Results** |
| --- | --- | --- | --- | --- |
| Embase | **#1** | 'leprosy'/exp OR 'hansen disease':ti,ab,kw OR lepros*:ti,ab,kw | 4.08.2025 | 38,145 |
|  | **#2** | 'tuberculosis'/exp OR tuberculosis:ti,ab,kw OR TB:ti,ab,kw |  | 417,576 |
|  | **#3** | 1 OR 2 |  | 451,423 |
|  | **#4** | 'gender identity'/exp OR 'sex characteristics'/exp OR 'sexual and gender minority'/exp OR gender:ti,ab,kw OR women:ti,ab,kw OR men:ti,ab,kw OR female:ti,ab,kw OR male:ti,ab,kw OR transgender*:ti,ab,kw OR transsexual*:ti,ab,kw OR intersex*:ti,ab,kw OR intergender:ti,ab,kw OR bisexual*:ti,ab,kw OR queer:ti,ab,kw OR homosexual*:ti,ab,kw OR lesbian*:ti,ab,kw OR "non-binary":ti,ab,kw OR nonbinary:ti,ab,kw OR sexual*:ti,ab,kw |  | 5,514,881 |
|  | **#5** | 3 AND 4 |  | 49,634 |
|  | **#6** | 'qualitative research'/exp OR 'interview'/exp OR 'focus group'/exp OR 'ethnology'/exp OR qualitative:ti,ab,kw OR interview*:ti,ab,kw OR "focus group*":ti,ab,kw OR ethnograph*:ti,ab,kw OR "grounded theory":ti,ab,kw OR narrative*:ti,ab,kw OR "thematic analysis":ti,ab,kw OR "content analysis":ti,ab,kw OR phenomenolog*:ti,ab,kw OR "life history":ti,ab,kw OR "semi-structured":ti,ab,kw OR "in-depth":ti,ab,kw OR "open-ended":ti,ab,kw OR "case study":ti,ab,kw OR "participant observation":ti,ab,kw OR autoethnograph*:ti,ab,kw |  | 1,596,959 |
|  | **#7** | "lived experience*":ti,ab,kw OR "life experience*":ti,ab,kw OR "patient experience*":ti,ab,kw OR "personal experience*":ti,ab,kw OR "illness experience*":ti,ab,kw OR experiences:ti,ab,kw OR experiential:ti,ab,kw OR "life-world":ti,ab,kw OR "daily life":ti,ab,kw OR "living with":ti,ab,kw OR stigma*:ti,ab,kw OR perceived:ti,ab,kw OR perception*:ti,ab,kw OR perspective*:ti,ab,kw OR understanding:ti,ab,kw OR journey:ti,ab,kw OR thematic:ti,ab,kw OR theme*:ti,ab,kw OR narrative*:ti,ab,kw OR narration:ti,ab,kw OR "life stor*":ti,ab,kw OR psychosocial:ti,ab,kw OR emotional:ti,ab,kw OR identity:ti,ab,kw OR meaning:ti,ab,kw |  | 4,114,293 |
|  | **#8** | 5 AND 6 AND 7 |  | 954 |
|  | **#9** | #8 NOT ('animal'/exp NOT 'human'/exp) |  | 953 |
|  | **#10** | #9 AND [1995-]/py |  | 947 |
