# Supplementary material for: Gendered experiences of people living with tuberculosis or leprosy: A global systematic review
Source: PLoS Negl Trop Dis. 2026 Sep 8;20(9):e0014711. doi: 10.1371/journal.pntd.0014711 (PMC13577493; doi:10.1371/journal.pntd.0014711)
Supplement: Supplementary file 3 [file pntd.0014711.s003.docx]

**S2 File**

**Data Extraction**

| **Study ID** | **Title** | **Year** | **Authors** | **Study Design** | **Disease** | **Country, Region** | **Gender** | **Demographics** | **Theme** |
| --- | --- | --- | --- | --- | --- | --- | --- | --- | --- |
| S_1 | Understanding stigma: The experiences of people with drug-sensitive pulmonary tuberculosis in Rawalpindi, Pakistan | 2025 | Ruby Stein, Anil Fastenau, Hina Aman, Nimer Ortuno-Guiterrez, Chris Schmotzer, Sophie CW Unterkircher, Eva Pilot | semi-structured interviews | TB (drug-sensitive pulmonary) | Pakistan, Rawalpindi | male and female | „11 female, 4 men, mostly married, mostly muslim, mostly housewifes, 18-64 years old“ | stigma |
| S_2 | Stigmatization and discrimination of female tuberculosis patients in Kyrgyzstan – a phenomenological study | 2025 | Rhea Brüggemann , Fabian Schlumberger3 , Firuza Chinshailo2 , Matthew Willis4* , Abdullaat Kadyrov2 , Gulmira Kalmambetova2 , Mo Chen1 , Sophie CW. Unterkircher3,4 , Nestan Moidunova2 , Altynai Sydykova and Anil Fastenau | phenomenological study | TB (lung, bone, DR, DR-sensitive, MDR, lymph, closed) | Kyrgistan, Bishkek | female | „19-59 years, mostly muslim, mostly married, mostly not working currently“ | stigma, consequences, mental Health, coping |
| S_3 | Using intersectional gender analysis to identify challenges in tuberculosis care at four health care facilities in Uganda | 2024 | Winters Muttamba1,2* , Samson Omongot1 , Irene Najjingo1 , Roseline Nuwarinda3 , Esther Buregyeya4 , Mariam Otmani del Barrio5 , Rosemary Morgan6 , Bruce Kirenga 1 and Sarah Ssali7 | cross-sectional stundy, gender seperated FGD | TB | Uganda, central (Buikewe, Kiboga, rural Nakaseke) | male and female | „A total of 17 FGDs were  conducted (9 for males and 8 for females) and included  53 men and 54 women, with each FGD having 4–8 par-ticipants.“ | risk of TB, TB knowledge, access to health information, health seeking behavior, masculinism, decision making power & autonomy |
| S_4 | Social isolation, social exclusion, and access to mental and tangible resources: mapping the gendered impact of tuberculosis-related stigma among men and women living with tuberculosis in Eastern Cape Province, South Africa | 2025 | Andrew Medina‑Marino, Lindsey de Vos, Joseph Daniels | Semi-Structured interviews | TB | South Africa, Eastern Cape Province | male and female | „142 men and women, median age 37 years, 86 were men, 72.1% of men unemployed, 29.0% of men living alone, 48.8% primary household breadwinner“ | experiences and perspectives of TB symptoms, access to clinical care and services, individual motivations for treatment, disclo- sure decision-making, social and familial network support during TB illness, perspectives of other men and women’s TB experiences, and probing for tangible and mental resources accessed during their TB illness and treatment journey |
| S_5 | Lived experiences of women suffering from Tuberculosis in Kashmir: an interpretative phenomenological study | 2025 | Rubeena Akhter 1 , Wakar Amin Zargar 2 and Fayaz Ahmad Paul 3 | interpretative phenomenological study | TB (extra-pulmonary, genital TB) | Pakistan, Kashmir | female | „21 married women, 15-49, rural & urban, Of the 21 study participants, 33% had studied up to secondary level, 23% had studied up to senior secondary, 19% had studied up to graduation, 4.7% had a master’s degree, and 19% could not read or write. Moreover, the study participants’ livelihoods and incomes ranged from holding private jobs to running their own home-based businesses. Among the 21 study participants, 47.6% were working in the private sector, 9.5% were government employees, 23.8% were doing home business, and 19% of them were housewives“ | infertility, reproductive concerns, reactions, mental health |
| S_6 | Gender Bias Issues Among Women Dropping Out of Tuberculosis Treatment in Batanghari Regency, Jambi, Indonesia | 2024 | Guspianto a,*, Dessy Pramudiani b , Ade Adriadi | in-depth interviews | TB | Indonesia, Batanghari Regency Jambi | female | „6 women who dropped out of treatment, 27-52 years, most housewives, low education“ | barriers to TB treatment comprising gender roles, stigma, health investment and nutrition, and perceptions of quality TB treatment services. |
| S_7 | A Glimpse of Present Day Perspectives of Stigma Associated with Leprosy: A Qualitative Study from a Tertiary Care Centre in Tezpur area of Assam, India | 2023 | L Sharma1, R Agarwal2, K Dadhich3, Y Dinker | cross-sectional prospective study, in-depth, FGD, qual. questionnaires | Leprosy | India, Tezpur area of Assam | male and female | „24 leprosy patients receiving MDT, 15 to 65 years, 16 males and 8 females, employed males and housewives, 4(16.66%) patients had deformities in the form of clawing of digits and foot drop, One patient had active leg ulcer who was undergoing regular silver dressings and weekly local infiltration of platelet rich plasma in the ulcer. Two patients were in type 2 reaction being treated with tablet thalidomide along with MB- MDT and 1 patient had type 1 reaction who was taking tapering doses of oral steroids and MB- MDT“ | knowledge, attitude and beliefs, social behaviours, quality of health services, access to resources, mental health, marriage, employment, decision making |
| S_8 | Why do patients with DR-TB do not complete their treatment? Findings of a qualitative study from Pakistan | 2024 | Muhammad Abubakar ,1,2 Matti Ullah,1 Muhammad Aamir Shaheen,3 Orva Abdullah | Semistructured, indepth, face- to- face interview | DR-TB (XDR &MDR) | Pakistan | male and female | „Loss-to-follow up (LTFU) patients, 14 XDR-TB, 25 MDR-TB, 21 male, 18 female, mean age of the participants was 35.92±11.17“ | loss-to-follow-up due to gender roles, financial dependancy, decision-making |
| S_9 | Factors affecting ability of TB patients to follow treatment guidelines – applying a capability approach | 2023 | B Aravind Chandru1 and Ravi Prasad Varma | follow-up in-depth interviews chosen from cross-sectional survey | TB | India, Thiruvananthapuram, Kerala | male and female | „4 women, 4 men, 20-60 years old, The immediate social context of the participants was primarily one of poverty and financial insecurity.“ | substance abuse for men, care-seeking, support, social roles |
| S_10 | A mixed methods study on men’s and women’s tuberculosis care journeys in Lusaka, Zambia—Implications for gender-tailored tuberculosis health promotion and case finding strategies | 2023 | Andrew D. KerkhoffID1*, Chanda Mwamba2, Jake M. Pry2,3, Mary Kagujje2, Sarah Nyangu2, Kondwelani Mateyo4, Nsala Sanjase2, Lophina ChilukutuID2, Katerina A. ChristopoulosID1, Monde Muyoyeta2, Anjali Sharma | in-depth interviews (IDIs), semi-structured guide, | TB | Zambia, Lusaka | male and female | „The 20 TB patients who participated in the IDIs had a median age of 44 years (range, 23–67), 10 (50%) were male and female, respectively, and 10 (50%) were persons with HIV, 8 (40%) persons without HIV and 2 (10%) had an unknown HIV status.“ | knowledge, care-seeking, making sense of disease, recommendations, delay, service quality, support |
| S_11 | Gender-related factors associated with delayed diagnosis of tuberculosis in Eastern Europe and Central Asia | 2022 | Nonna Turusbekova1*, Cristina Celan2, Liliana Caraulan3, Oxana Rucsineanu4, Mariam Jibuti5, Oxana Ibragimova6 and Nargis Saidova7 | in-depth interviews and focus group discussions | TB | Eastern Europe, Central Asia (Georgia, Kazakhtan, Moldova, Tajikistan) | male and female | „86 in-depth interview respondents and 21 focus groups (227 participants). Except for Kazakhstan, where there was an equal number of men and women among the respondents, in other countries there were more men“ | gender-related barriers to symptom recognition, care seeking, and obtaining the correct diagnosis. |
| S_12 | The influence of leprosy and other disabilities on marital relationships and sexual health among married women in Indonesia: A qualitative study into experiences and coping | 2022 | Dennise F. P. Susantoa, †, Anna T. van ‘t Noordendea, †, Eriando R. Septianb, Wim H. van Brakela, Ruth M. H. Petersc & I. Irwanto | ross-sectional comparative study design with a qualitative approach; semi-structured in-depth interview | Leprosy and disabilities | Indonesia, Tegal regency | female | „9 women, 21-42 years, 8 married, 1 divorces, mostly housewives, mostly grade 1 disability, treatment mostly completed“ | feelings, knowledge, disclosing, coping, relationship and autonomy, support, gender roles, marriage and sexual health |
| S_13 | Perceptions, Attitudes, Experiences and Opinions of Tuberculosis Associated Stigma: A Qualitative Study of the Perspectives among the Bolgatanga Municipality People of Ghana | 2022 | K. A. T. M. Ehsanul Huq 1,* , Michiko Moriyama 1 , David Krause 2, Habiba Shirin 1 , John Koku Awoonor-Willaims 3, Mahfuzur Rahman 4 and Md Moshiur Rahman | FGDs seperated by gender | TB | Bolgatanga, Ghana | male and female | „9 participants (also includes those not affected), 10 males and 9 females between the ages of 27 and 55 years old. Five partic- ipants had the highest educational level of junior secondary school. Seven participants had some form of education, at least up to primary-three levels and another seven participants had no education. Most of the respondents (eleven participants) were farmers, and the remaining eight participants were traders. Regarding religion, nine of the participants were Traditionalists; they traditionally expressed their belief in a supreme God as Nyame or Mawu [29], five were Christians, and five were Muslims“ | support of friends and family, effect on marriage of their children, economic effects, treatment adherence |
| S_14 | Qualitative Identification of Intervention Preferences to Support Men’s Engagement and Retention in TB Care in South Africa | 2022 | Andrew Medina-Marino1,2,3* , Dana Bezuidenhout3,4* , Nondumiso Ngcelwane5 , Morna Cornell6 , Milton Wainberg7,8 , Chris Beyrer9,10 , Linda-Gail Bekker10 , and Joseph Daniels | exploratory study, IDIs | TB | South Africa, Eastern Cape | male | „30 men, 23-60 years“ | treatment adherence, mental resources, support, men socializing, male roles, education/knowledge |
| S_15 | Pre-Diagnosis Health Seeking Behaviors and Experiences Post-Diagnosis, among Men Diagnosed with Tuberculosis in a District of Gauteng Metropolitan City, South Africa: In-Depth Interviews | 2022 | Sewele Makgopa, Lindiwe P. Cele * and Mathildah M. Mokgatle | exploratory qualitative study, using in-depth interviews (IDIs) | TB | South Africa, District of Gauteng Metropolitan City | male | „25 participants, 18 to 61 years with most (8/18) between the ages of 32 and 41 years. Most were South African (15/22), Sotho speaking (4/12), belonging to the Christian religion (12/18) and living in a shack (15/19). Only 5/19 and 3/19, respectively, were married and in steady relationships. The socioeconomic data revealed that nearly half were unemployed (8/17), while 6/15 had a regular salary, with another 6/15 relying on handouts. Most weighed less than 70 kg (17/20) and only (4/12) had family support. The majority had between one (1) and four (4) children (11/15) with half (7/14) having between grades 8 and 11 level of education“ | healthcare-seeking, delay, beliefs/reactions (making sense of disease), disclosure, stigma, fears, support, daily live changes, lessons learned |
| S_16 | The Impact of Leprosy and Physical Disability on Marital and Sexual Relationships of Married Nepali Men | 2022 | Maartje J. Meis1* , Anna Tiny van’t Noordende1, Liesbeth F. Mieras 1 , Nand Lal Banstola2, Krishna Dhakal 2 , Dirk R. Essink3, Wim H. van Brakel | cross-sectional design with a qualitative approach,semi-structured interviews and focus group discussions (FGDs) | Leprosy | Nepal, Morang District | male | „10 men, all married, 21-42 years, mostly no to low education, employed (7/3), hinduism, rural and urban(5/5), arranged marriage and love (7/3), Of the men affected by leprosy, 5 had a G1D and 5 had a G2D, The median time since diagnosis was four years.“ | marrital relationships, discrimination, sexual relationship |
| S_17 | Improving pathways to care through interventions cocreated with communities: a qualitative investigation of men's barriers to tuberculosis care-seeking in an informal settlement in Blantyre, Malawi | 2021 | Mackwellings Maganizo Phiri ,1 Effie Makepeace,2 Margaret Nyali,3 Moses Kumwenda, 1,3,4 Elizabeth Corbett,5 Katherine Fielding,6 Augustine Choko,4 Peter MacPherson, 4,7 Eleanor Elizabeth MacPherson | qualitative study design (in-depth interviews (IDIs)) and participatory workshops (PWs) | TB | Malawi, Blantyre | male and female | „30 participants, 15 men, 15 women, 18-35 years (15), 35 and above (15), mostly no school/primary level (22), 27 employed“ | health-seeking, socio-economic conditions/responsibilites, gendered social norms/power relaations, household, biomedical care vs complementary care, health system constraints, recommendations |
| S_18 | Women and leprosy: Interferences and experiences | 2021 | Marcela Gonçalves, Karen da Silva Santos, Simoe Santana da Silva, Thalita Caroline Cardoso Marcussi, Kisa Valladao Carvalho, Cinira Magali Fortuna | descriptive study with a qualitative approach, cartographic research | Leprosy | Brazil, Sao Paulo | female | „9 women, 30-62 years old, 4 married, one seperated, one divorces, two in stable relationship, one single, three were away from work, one retired, one was a housewife, one a cleaning lady, one an epilator, one a seamstress and one a day care worker. Two women had completed elementary school, two had incomplete elementary school, four had completed high school and only one had completed higher education.“ | looks and appearance, earning a living, violence and power |
| S_19 | Masculinity, resources, and retention in care: South African men's behaviors and experiences while engaged in TB care and treatment | 2021 | Joseph Daniels a,1,*, Andrew Medina-Marino b,c,d,1,**, Katherine Glockner b, Emily Grew e, Nondumiso Ngcelwane, Aaron Kipp | semi-structured interviews | TB | South Africa, Eastern Cape, Buffalo City | male | „31 men, 23-60 years, A total of 16 participants were currently engaged in treatment and 15 had recently completed treatment. Among those 31 men, 11 discussed having temporarily stopped treatment in the past and 1 described cycling in and out of treatment. Most men were unemployed or worked in the informal sector as day laborers.“ | economic insecurities, barriers to care, social norms and expectations of men, health-seeking, stigma, support, fatherhood, |
| S_20 | Challenges in accessing and utilising health services for women accessing DOTS TB services in Kolkata, India | 2020 | Reshmi Mukerjia,b and Janet M. Turan | qualitative exploration, IDI | TB | India, Kolkata | female | „20 women, 80% of participants were Muslim, all came from low socioeconomic backgrounds, and 50% were married. 70% of the participants were currently on TB treatment and 35% had been treated for TB prior to this episode“ | DOTS/care challenges, quality of care, gender roles, resource constraints |
| S_21 | Disease experiences of female patients with Hansen's disease residing in settlement in Korea | 2020 | Ho Gi Jung and Ya Ki Yang | inductive phenomenological study | Leprosy | South Korea, Sorok Island (settlement villages) | female | „11 women, mean age 75 years, all married, seven still living with spouse, four as widows, hospitalized in Sorokdo hospital and Aeyangwon, but were subsequently relocated to settlement villages after recovering and testing negative. Their age at the time of relocation ranged between mini- mum of 28 years and maximum of 45 years. The length of stay at settlement villages varied between minimum of 20 years and maximum of 60 years“ | changing body, isolation, quality of care, parental/women rights, lost feminity and motherhood, delay, (mental health) |
| S_22 | Burden of stigma among tuberculosis patients in a pastoralist community in Kenya: A mixed methods study | 2020 | Grace Wambura MbuthiaI, Henry D. N. Nyamogoba2, Silvia S. Chiang3,4, Stephen T. McGarvey | explanatory sequential mixed methods approach, in-depth interviews and FGD | TB | Kenya, West Pokot County | male and female | „61 participants, 15 IDI (7 women, 8 men), 46 FGD (22 women, 24 men), 27-61 years old“ | stigma, isolation |
| S_23 | Adaptive strategy of women's leprosy in indonesia psychic experience of women with leprosy in living a community life | 2020 | Abd. Nasir*1, Ah Yusuf1, Muhammad Yulianto Listiawan1, Susilo Harianto1, Nuruddin1, Nuh Huda | qualitative phenomenology study | Leprosy | Indonesia, east (Surabaya) | female | „17 women, 14-65 years old“ | mental stress, shame, discrimination, exclusion, appearance, religious belief as coping |
| S_24 | Disclosure of disease among women affected by leprosy: A qualitative study | 2020 | Senthil Kumar.R, Archana Kumar, Pitchaimani Govindharaj, Suganya Paneerselvam | qualitative semi-structured (questionnaire) further information missing | Leprosy | India, Champa, Chhattisgarh State | female | „Of the 57 women, 20 (35%) were between 18 and 30 years of age, 88% were married and 51% were illiterate. Nearly half of them (45%) were housewives and 84% had family income below Rs 5000 (Indian currency), About three-fourths (74%) of the affected women were diagnosed as multibacillary disease and 23% had grade 2 disability. Nearly half of them (44%) had been released from treatment (RFT) for more than 2 years, and 30% came to the hospital for illness due to leprosy“ | fears, disclosure, reactions, shame, discrimination, stigma |
| S_25 | “Who Will Marry a Diseased Girl?” Marriage, Gender, and Tuberculosis Stigma in Asia | 2019 | Bethan Hatherall1 , James N. Newell2 , Nick Emmel2 , Sushil Chandra Baral3 , and Muhammad Amir Khan | grounded theory qualitative study, IDI, FGD | TB | Bangladesh, West Shilmandi (Norshingdi district), Pakistan, Bagga Sheikhan & Sultan Pura (Rawalpindi), Nepal, Godawari & Badikhel Village (Lalitpur district and submetropolitan city) | male and female | „FGD three (two with women and one with men) were comprised specifically of people with TB“ | marriage prospects, gender roles and responsibilites, motherhood/pregnancy, disclosure/concealment |
| S_26 | Representations of leprosy's stigma in the women of the Jequitinhonha Valley-MG | 2019 | Ricardo Jardim Neiva, Marcia Grisotti | narrative interview study | Leprosy | Brazil, Jequitinhonha Valley-MG | female | „8 women, 18-62, wo were retired, two were artisans, two were formal workers, o n e was unemployed and seeking re-employment at the time of the interview, and one was self-declared as a “housewife.“ | disclosure/concealment, religion, trust in HCW and people, knowledge |
| S_27 | Exploring manifestations of tb-related stigma experienced by women in Kolkata, India | 2018 | Reshmi Mukerji and Janet M. Turan | IDI interviews | TB | India, Kolkata | female | „20 women, 80% muslim, The majority of participants were young adults aged 18–24 (50%) and predominantly Muslim (80%), with an equal split between married and single individuals. Most had secondary education (45%), low household income below $100 (78%), and were mainly housewives or employed in informal work (30% each). Regarding TB, 70% were currently on treatment, 70% had pulmonary TB, and 65% were being treated for the first time.“ | stigma, discrimination, marriage and relationship, disclosure, shame, mental health, fears, coping, |
| S_28 | Tuberculosis and the sexual and reproductive lives of women in Bangladesh | 2018 | Mrittika Barua, Francien Van Driel, Willy Jansen | IDI | TB | Bangladesh, Narsingdi and Dhaka | female | „9 women, mostly 25-30 years, mostly housewives, married with children, living with husbands, one had never attended school, three had a primary education, three had one or more years of secondary education, and one had finished her secondary education (passed her Second School Certificate exam) and one was not asked. All women were in their first marriages except for one who was in her second marriage. Except for the three women who had been married less than a year at the time of the interview, the rest had at least one child. All patients with children had at least one son, although one reported having a son from her previous marriage. The lowest family income reported was 8000 BDT (US$96) and the highest was 20,000 BDT (US$240) per month. This range is considered a low income for standard living“ | gender and relationships, support, abuse, motherhood, pregnancy, reproductive, sexual lives |
| S_29 | The reaction is the most difficult, it is worse than leprosy: Contradictions and ambiguities in the experience of women with Leprosy reactions | 2018 | Lidiane Mara de Ávila e Silva, Reni Aparecida Barsaglini | semi-structured IDI | Leprosy | Brazil, Cuiabá | female | „7 women, 26-55, four of whom are married, two separated and one divorced. They declare themselves to be white (three), brown (three) and black (one). There are similarities between the contexts and spaces, because most of them are from low-income families and live on half the minimum wage per person, on average. As for infrastructure services, such as water, for example, all interviewees reported having access to piped water.most families live on unpaved streets and deal with puddles and open sewers. The houses have few rooms, with one or two bedrooms at most, and some have no flooring, paint or gates, n terms of education, women have an average of eight years of schooling, and most work in jobs that do not typically require specific training, such as cleaning, childcare (nannying) and cooking.,all christian“ | care-seeking, gender roles, delay, reaction, knowledge/making sense, appearance, trust in treatment |
| S_30 | Work and Leprosy: women in their pains, struggles and toils | 2017 | Marcela Gonçalves I , Michely Aline Rodrigues do PradoI , Simone Santana da SilvaII , Karen da Silva Santos II , Priscila Norié de AraujoII , Cinira Magali Fortuna | qualitative, explorative study, FGD | Leprosy | Brazil, Ribeirão Preto, São Paulo | female | „10 women, aged between 24 to 74 years old, among whom 2 were married, 5 were single, 1 was a widow and 1 had a stable union. Regarding schooling, most of the participants had completed high school, only one woman did not finish high school, two of the study par- ticipants attended elementary school and one had a postgraduate level. Among the work activities developed by the study partici- pants were: a telephone operator, two cash operators, a produc- tion assistant, a day laborer, a general service worker, a biochemi- cal pharmacist, a retiree and one profession is kitchen assistant).“ | physical pain, fear, shame, effects on work and daily tasks, gender roles, moterhood |
| S_31 | 'It makes the patient's spirit weaker': Tuberculosis stigma and gender interaction in Dar es Salaam, Tanzania | 2017 | C. Miller,* † J. Huston,‡ L. Samu,§ S. Mfinanga,§ P. Hopewell,* † E. Fair | FGD | TB | Tanzania, Dar es Salaam, Kinondoni District (urban) | male and female | „24 TB patients, four FGD with six participants each“ | gendered stigma, beliefs, relationship, marriage, social roles, daily effects |
| S_32 | Where there is hope: A qualitative study examining patients' adherence to multi-drug resistant tuberculosis treatment in Karakalpakstan, Uzbekistan | 2016 | Shona Horter 1* , Beverley Stringer 1 , Jane Greig 1 , Akhmet Amangeldiev 2 , Mirzagaleb N. Tillashaikhov 3 , Nargiza Parpieva 3 , Zinaida Tigay 4 and Philipp du Cros | IDI | TB (MDR) | Uzbekistan, Karakalpakstan | male and female | „35 patients, 54% female, 46% male, 16-69 years, 34% no prior treatment history, 15 ‘adherent’, 12 ‘partially adherent’, and 8 LFT patients“ | gender roles and expectations, treatment adherence, support, socio-economic barriers for men |
| S_33 | A disease called stigma: the experience of stigma among African men with TB diagnosis in London | 2017 | M.J. Chinouya, O. Adeyanju | semi-structured interviews | TB | England, London | male | „10 men, born in different African countries including Zimbabwe (N ¼ 3), Uganda (N ¼ 3), Nigeria (N ¼ 2), Ghana (N ¼ 1) and Sudan (N ¼ 1). Mostly (N ¼ 8) were unemployed and a few (N ¼ 3) had been in immigration detention centres. Their age ranged from 33 to 67 years old, all of them have been living in the UK for less than 15 years.“ | gender role, delay/care-seeking/self-diagnosis, stigma, isolation |
| S_34 | Mexican American Men’s Experience of Living With Tuberculosis on the U.S.–Mexico Border | 2016 | Julie Ann Zuñiga, PhD, RN1 , Silvia Muñoz, PhD, FNP2 , Mary Zuñiga Johnson, MS2 , and Alexandra A. García, PhD, RN, FAAN | This qualitative descriptive study was a secondary analy- sis of a larger qualitative (hermeneutic phenomenological) study | TB | US-Mexico Border | male | „13 men, All of the participants were Mexican Americans living in the United States at the time of the interview, although some of the men stated that they were born in Mexico but had recently immigrated to the United States. About half of the men were employed, working in trucking, migrant farming, food services, and construction; however, only one participant was able to work continuously during TB treatment. He also was the most educated, with an associ- ate’s degree. The average number of years of education was 8.5 (range of 0-13 years), with one man having no formal (school) education. The average number of per- sons living in the household was nearly four.“ | delay, knowledge, depression, disclosure, isolation, stigma |
| S_35 | The impact of leprosy on marital relationships and sexual health among married women in eastern Nepal | 2016 | Anna T. van ’t Noordende, 1 Wim H. van Brakel,1,2 Nandlal Banstola, 3 and Krishna P. Dhakal | cross-sectional, nonran- dom survey design with a qualitative approach | Leprosy | Nepal, Eastern Terai | female | „10 women, 6 had a grade 1 impairment and 4 had a grade 2 impairment, 22-50 years (35 years mean age), all married, majority hindu, lived in rural areas, most marriages arranged, mostly with children, more than half didnt work,“ | knowledge, disclosure, marriage/relationship/sex, abuse by husband |
| S_36 | Barriers to completing TB diagnosis in Yemen: Services should respond to patients' needs | 2014 | Rachel M. Anderson de Cuevas 1*, Najla Al-Sonboli2 , Nasher Al-Aghbari2 , Mohammed A. Yassin 1 , Luis E. Cuevas 3 , Sally J. Theobald | IDIs, FGD | TB | Yemen, Sana'a | male and female | „16 adults who had completed and 11 who had abandoned the diagnostic process, and 20 who had registered for treatment. Participants completing diagnosis com- prised 6 females and 10 males, aged 22 to 60 years, of whom 13 were married. Seven participants resided outside Sana’a. Three females and 5 males were working and 4 participants had schooling. Participants abandoning the diagnosis comprised 3 females and 8 males, aged 20 to 83 years, of whom 6 were married. One participant resided outside Sana’a. None of the females and 5 males were working and 8 participants had schooling. Participants who had registered for treatment com- prised 13 females and 7 males, aged 18 to 50 years.“ | support, women's access to care, gender roles and expectations |
| S_37 | Socio-cultural influences on adherence to tuberculosis treatment in rural India | 2014 | Rubina Shiotani and Monique Hennink | IDI | TB | India, Gujarat state | male and female | „The study included 21 tuberculosis (TB) patients, most of whom were between 30 and 49 years old (12 patients) and almost evenly split by gender (11 males and 10 females). All participants were married, with 10 belonging to scheduled tribes and 11 to non-tribal groups; 16 patients lived below the poverty line. Nearly half (10) were engaged in house work, most received treatment at the Jhagadia Primary Health Centre (10), and the largest groups were those cured (7) or who had completed treatment (6).“ | economic barriers, social stigma, marriage, reputation |
| S_38 | Gender differences in the interpretation of experiences of patients with tuberculosis in Medellín, Colombia | 2015 | Liliana Villa Vélez1 María Patricia Arbeláez Montoya | semi-structured interviews | TB | Colombia, Valle de Aburr, Medellin | male and female | „12 men and women, Men reported a mean age of 43 years (interquartile range: 26 - 52) and women reported a mean age of 37 years (interquartile range: 27 - 39).“ | disease expression, delay, knowledge, fear, support, isolation, shame, trust in HCW |
| S_39 | Control, struggle, and emergent masculinities: A qualitative study of men's care-seeking determinants for chronic cough and tuberculosis symptoms in Blantyre, Malawi | 2014 | Jeremiah Chikovore 1,4* , Graham Hart 2 , Moses Kumwenda 3,4 , Geoffrey A Chipungu 3 , Nicola Desmond and Liz Corbett | pre-intervention exploratory study;IDI | TB | Malawi, Blantyre | male | „20 recently diagnosed patients, 14 women, six men“ | social gender roles, (threats to) masculinity, economic barriers, care behavior |
| S_40 | Narratives around concealment and agency for stigmareduction: A study of women affected by leprosy in Cirebon District, Indonesia | 2014 | Ruth M H Peters 1*, Miranda E Hofker1, Wim H van Brakel 2, Marjolein B M Zweekhorst1, Francisia S S E Seda 3, Irwanto 3, Joske F G Bunders | ualitative semi- structured in-depth interviews | Leprosy | Indonesia, Cirebon District | female | „53 women, 16-80 years old, 33 married, though some said that their current marriage was not their first, 9 were single, 1 was engaged, and 10 were widows. Most of the women were housewives and/or caregivers, 36 (68%) had children, and some were employed, for instance as shopkeepers, farmers, tailors, teachers or domestic workers. Most women had finished the 6 or 12 months of multi-drug therapy for leprosy. At the time of the interview, only 14 were still in treatment. Eight had a leprosy-related impairment to hands, feet and/or eyes“ | disclosure, support, appearence, social stigma, self-stigma, coping |
| S_41 | Understanding the Gender Aspects of Tuberculosis: A Narrative Analysis of the Lived Experiences of Women With TB in Slums of Delhi, India | 2012 | Koushambhi Basu Khan | ethnographic design, short structured interviews with pa- tients 1 diagnosed with pulmonary tuberculosis, as well as follow up in-depth interviews with a select group of patient | TB | India, Slums of Delhi | male and female | „15 women, 10 men“ | beliefs/knowledge, marriage prospects, discrimination, treatment-seeking/adherence, autonomy, gender roles, limited resources |
| S_42 | The body i was and the body i am: Conceptions of women with alterations caused by leprosy | 2010 | Iací Proença Palmeira2 , Márcia de Assunção Ferreira | qualitative, exploratory and descriptive | Leprosy | Brazil, Rio de Janeiro | female | „43 women with bodily changes, between 18 and 59 years old and their ages were stratified in three age groups, divided as follows: 18-29 years; 30-41 years; and 42-59 years. Most of the women (19), i.e. 43%, were between 30 and 41 years old. No difference was found between the other groups, each of which contained 12 subjects“ | appearance, bodily changes, self-image, coping |
| S_43 | A qualitative study exploring delayed diagnosis and stigmatisation of tuberculosis amongst women in Uganda | 2012 | Laura Macfarlane, James N. Newell | semi-structured interviews | TB | Uganda, Kampala, Kawempe Division | female | „12 women, delayed seeking, sputum-positive TB“ | delay, stigma, disclosure, financial barriers |
| S_44 | Filipino Women's Tuberculosis Care Seeking Experience in an Urban Poor Setting: A Socioecological Perspective | 2012 | Alice Hu, Evelyn Loo, Peter J Winch, Pamela J Surkan | in-depth interviews (IDIs) and focus group discussions (FGDs). | TB | Phillippines, Paytas (urban poor) | female | „13 women, five received care from LGUs and eight from NGOs. The age of the women ranged from 23 to 73 years old. Four of the 13 women were older than 60. All were married or had a partner. The mothers had between one and seven children, while two women had no children of their own. Almost all participants worked in the informal sector (N = 7), for example, as scavengers and pillowmakers. Only two women worked in the formal sector prior to contracting TB, and the others had no defined work. In our sampling, we also included a range of patient types: symptomatics who were referred to TB diagnostics (N = 2), newly enrolled (N = 6), returning, defaulted, or both (N = 5) TB patients.“ | delay, care-seeking, knowledge/understanding, fears, gender roles and responsibilites, economic barriers, motherhood, support, stigma/discrimination, health system |
| S_45 | Gender-related factors influencing tuberculosis control in shantytowns: A qualitative study | 2010 | Dami A Onifade, Angela M Bayer1, Rosario Montoya 1,3, Marie Haro1,3, Jessica Alva1,3, Jessica Franco 1,3, Rosario Sosa 1,3, Betty Valiente1,3, Enit Valera 1,3, Carolyn M Ford1, Colleen D Acosta1 and Carlton A Evans | semi-structured interviews and focus group discussions | TB | Peru, Ventanilla (shantytowns) | male and female | „12 IDI with 6 male patients: (mea9n age 34, 17-6), 1 unemployed, 5 informal irregular work, 8 female patients: (mean:34, 17-52), 6 unemployed, 1 cook, 1 entrepreneur // FGD 6 male patients (mean: 25, 18-31), 6 female (mean:31, 19-52)“ | gender roles, barriers to care, low priority women's health, autonomy, treatment-seeking, discrimination, knowledge, healthcare-service quality |
| S_46 | Differences in the adherence to tuberculosis treatment in relation to sex in the health district of Freguesia do Ó/ Brasilândia - São Paulo | 2010 | Pericles Nogueira, Raquel Queiroz | semi-structured interviews | TB | Brazil, Sao Paulo, Freguesia do O | male and female | „28 patients, 11 males, 17 females, 18-29 years old, women more educated than men (77,8 % completed secondary education, only 22.22% of the men,“ | knowledge, appearance, reaction, adherence, |
| S_47 | From contagious to chronic: A life course experience with leprosy in Taiwanese women | 2006 | CAROL SHIEH*, HSIU-HUNG WANG** , þ & CHING-FENG LIN | narrative analysis | Leprosy | Taiwan, Lo-Sheng leprosarium and a leprosy long- term care centre (LLTCC) (north and south) | female | „21 women, 12 lived in the leprosarium, five lived in the LLTCC, and another four were community-dwelling women who were former patients of the LLTCC. These four women had stayed in the centre from 6 months to 3 years and still utilized the centre for leprosy follow-ups after discharge. The rest of the women had lived in either the leprosarium for 30 – 64 years or the LLTCC for 4 –16 years at the time of this study. The mean age of these 21 participants was 67.7 years old, ranging from 54 to 88. Six of the 21 women (28%) had no formal education, 13 (62%) had either an elementary education or had attended school for 2 or 3 years, and 2 (10%) had received a middle school education. Only one woman in this study had a regular, paid job outside her home and she was from the community-dwelling group. At the time of the study, 12 (57%) were married, 8 (38%) were widows, and one (5%) was divorced. Twelve (57%) women currently married or had married a man who also had leprosy. Of these 12 women, 10 (83%) were from the Lo-Sheng leprosarium. Three (14%) of the women had never given birth, although one of these three women had adopted a son. Most of the women (n ¼ 15, 71%) had acquired their leprosy symptoms during childhood or adolescence, five (24%) during young adulthood (20– 30 years old), and one (5%) in her 40s. The degree of physical impairments and disabilities related to leprosy varied from skin scars, to disfigured faces, blindness, claw fingers, drop feet, and amputated legs. The information regarding MDT (ever received, the date it began, and the length of MDT treatment) for each woman was not collected because data were either not available or incomplete. None of the 21 women was on any leprosy medication at the time of the study. The age, education, marital status, and physical impairments and disabilities were similar in the women of Lo-Sheng leprosarium, LLTCC, and the community-dwelling group. One major difference was that all of the women in the Lo-Sheng leprosarium were admitted before MDT was recommended in 1982“ | appearance, stigma, shame, treatment-seeking, motherhood, marriage, daily struggles |
| S_48 | Fear and social isolation as consequences of tuberculosis in Vietnam: A gender analysis | 2001 | Nguyen Hoang Long 1 a b d, Eva Johansson b d, Vinod K Diwan b c, Anna Winkvist | FGD | TB | Vietnam, Hanoi, Quang Ninh, Ho Chi Minh City, Quang Nam-Da Nang | male and female | „8-10 per group, 4 FGD, two only men, two only women, no further information“ | knowledge, reaction, economic burden, isolation/exclusion, marriage, adherence |
| S_49 | Trust and transparency in human encounters in tuberculosis control: Lessons learned from Vietnam | 2002 | Eva Johansson, Anna Wikvist | qualitative | TB | Vietnam, Quang Ninh Province | male and female | „24 patients (15 males, 9 females) 17-74 years old, wide variety of employment (reitred to adolescents still in school)“ | gender roles, reactions, stigma, isolation, treatment adherence, knowledge/understanding |
| S_50 | Tuberculosis in Bombay: New insights from poor urban patients | 1997 | DINESH M NAIR, ANNIE GEORGE,AND K T CHACK0 | IDI | TB | India, Bombay | male and female | „16 patients, 4 married men, 3 single men, 5 married women, 4 single women“ | economic burden, support, disclosure, worry |
| S_51 | Double jeopardy: women and leprosy in India. | 2015 | Carol Vlassoff, Seemantinee Khot, Shoba Rao | IDI | Leprosy | India, Bihar and Maharashtra | female | „25 patients (13 males, 12 females), no further information“ | rejection/exclusion, marriage/prospects, knowledge |
| S_52 | Life Experiences of Women with Leprosy in South Korea | 2020 | Hogi Jung, Rayun Yang, Yaki Yang | phenomenological method | Leprosy | South Kora, Sorokdo | female | „The average age of the study participants was 78 years, and all of them had experience of marriage after hospitalization in Sorokdo Hospital. After bereavement, there are two people who live alone and five who live with their spouse. The birth place is Jeollanam-do 3, Jeollabuk-do 2, Chungcheongbuk-do 2, which seems to be related to the Sorokdo Hospital located in Jeollanamdo. The pathway of hospital admission to Sorokdo Hospital is diverse. The hospitalization route was confirmed by the invitation of the family, the hospitalization through the protection center, and the forced acceptance, it can be seen that the hospitalization procedure of the Sorokdo Hospital was not formulated at that time. At the time of admission, the minimum age is 16 and the maximum age is 28. The period of living in Sorokdo Hospital varies from 38 years to 67 years. Participants can see that they have lived in Sorokdo Hospital for a very long time, including the critical period of their lives.“ | appearance, shame, isolatiom, marriages, motherhood |
| S_53 | Lived experiences of female patients aged 15-49 years undergoing treatment for multi-drug resistant tuberculosis (MDR-TB) in Lira Regional Referral Hospital, Uganda | 2024 | Kizito Omona & Christopher Ogwang | phenomenological design | MDR-TB | Uganda, Lira | female | „15 patients, 2-36 months of treatement (mean:9), 15-60 years old (mean: 34,9), 13 unemployed farmers, six of whom catholic, six completed primary education, 12 married“ | support, discrimination, stigma, treatment-seeking, economic barriers |
| S_54 | Tuberculosis in Pakistan: Socio-cultural constraints and opportunities in treatment | 2000 | Amir Khan a, b , John Walley b , James Newell b, *, Naghma Imdad | in-depth semi structured interviews | TB | Pakistan, Rawalpindi; Sahiwal and Gujranwala | male and female | „36 interviewees, 30 ere in the economi- cally active age range of 18±59 years. Nineteen were married. Most respondents lived in relatively large families: seven with a household size of seven, 28 of between eight and 18, and one of 23. Family size was generally smaller among rural interviewees than among those living in urban areas. Most respondents lived in a house having between one and three rooms, with those from urban areas having fewer rooms. Defaulters came from the most overcrowded families with 3.8 persons/room, while completers were some- what less overcrowded, with 3.1 persons/room. Thirteen of the 36 respondents had only one mem- ber of the household who was wage earning: only one respondent's household had no earning member and relied on begging and charity. Six of the respondents' households had only male earners, Æve had both male and female earners, and two only female earners. The average number of earning members per household was higher among households of respondents from urban areas than those from rural areas (respectively two and 1.2 earners per household). All respondents reported very low household incomes, with all but Æve having incomes below the recognised poverty level of 3745 Pakistani rupees (World Bank, 1998). The average household income reported by respondents from rural families was 1870 rupees: the corresponding income among urban families was 3210 rupees. Respondents who had com- pleted treatment had lower both average and per capita monthly household incomes than other groups (see Table 2). Seventeen of the respondents were literate, the lit- eracy rate being much higher among men (12/18) and among urban respondents (12/18). Among completers, midway patients and defaulters respectively, the aver- age number of literates in respondents' families were 4.2, 2.0 and 2.3.“ | treatment-seeking, stigma |
| S_55 | Perception and social consequences of tuberculosis: A focus group study of tuberculosis patients in Sialkot, Pakistan | 1995 | R. Liefooghe, N. Michiels, S. Habib, M.B. Moran and A. de Muynck | FGD divided by gender | TB | Pakistan, Sialkot | male and female | „48 patients, 3 male and 3 female FGD, mean age 35 years, females were younger than males (40 yr / 15.7 yr) vs. females (29 yr/ 13.2 yr), 86% muslim, 14& christian, 72% new patients, 14% relapsed, 14% readmitted“ | economic burden, marriage prospects, pregnancy |
| S_56 | Marks on oneself: experiencing the pain of (self) preconception | 2013 | Iací Proença PalmeiraI , Ana Beatriz Azevedo QueirozII , Márcia de Assunção Ferreira | qualitative research, using Social Representation Theory (SRT), semi-structured interivews | Leprosy | Brazil, Marituba/Pará (former leprosy colony Colônia de Marituba | female | „43 women with bodily changes caused by leprosy, The majority (37.21%) of the women in this study were aged between 29 and 38 years; more than half (53.81%) lived in Marituba; 53.50% were Catholic; 34.89% had not completed primary education; 58.14% had a monthly income ranging from 1 to 2 minimum wages, and 58.14% were exclusively engaged in domestic chores. The clinical therapeutic profile of the women showed a group of women who were mostly multibacillary (79.07%); with zero degree of physical disability (53.49%); with reactive episodes (62.79%); and highly cured (69.77%), but remained linked to the URE for other treatments.“ | appearance, social representations, stigma, understanding, coping |
| S_57 | Gender differences in the social support network among lung tuberculosis patients: Qualitative study in two communities of the state of Veracruz, Mexico | 2004 | Hernández, C.R. | IDI | TB (lung) | Mexico, Veracruz; Orizaba and Tuxapanguillo | male and female | „A total of 16 TB patients were interviewed: 10 from Orizaba (8 men, 2 women) and 6 from Tuxpanguillo (1 man, 5 women). In Orizaba, participants were 18–68 years old, mostly mestizo (7 men and 2 women) with one Nahua man who identified as “urban.” They had low formal education, having started work young, and held diverse occupations. Their cement homes showed severe humidity and poor ventilation. In Tuxpanguillo, all patients were Indigenous Nahuas aged 19–52, bilingual in Spanish and Nahuatl, and worked as day laborers earning about 50 centavos per kilo of coffee harvested. Compared to Orizaba residents, they faced higher illiteracy, overcrowding, lack of services, and greater social marginalization, though both groups shared similar age ranges and marital diversity.“ | support, care-seeking, gender roles |
