# Supplementary material for: Gendered experiences of people living with tuberculosis or leprosy: A global systematic review
Source: PLoS Negl Trop Dis. 2026 Sep 8;20(9):e0014711. doi: 10.1371/journal.pntd.0014711 (PMC13577493; doi:10.1371/journal.pntd.0014711)
Supplement: Supplementary file 4 [file pntd.0014711.s004.docx]

**S3 File**

**Synthesis of Study Results**

*Theme 1*

| **Theme 1: Quality of Care** | | |
| --- | --- | --- |
| ***Negative Experiences*** | | |
| **Study ID** | **Gender & Disease** |  |
|  | **Leprosy** |  |
| 7 | mw | non-availability of medicines |
| 7 | m | long waiting hours at OPD |
| 7, 29, 47 | w | long waiting time for diagnosis (months-years) |
| 29, 47 |  | information by health services didnt include information on reactions, disabilites, med side effects (overdosis with pills after community centres gave them pills) |
| 21 |  | uncomfortable hospitals (prison-like), *They used to say if you’re going to die, die on a Saturday since they don’t do autopsies on Sundays. When we die, they always do an autopsy, so if you just want to be cremated, then just die on Saturday. (#2)* |
| 40, 47 |  | HCW disclosed disease to community, visited home |
| 47, 23 |  | wrong diagnosis |
|  | **TB** |  |
| 10 | mw | delay of diagnosis |
| 17 |  | bad treatment from HCW *(They are harsh and treat you as though you were not humans. Some of them go out to chat to each other, where they laugh and do things of their own. Like today, as we were collecting drugs, one of them said, “You men are looking at me! You should look down. Why are you looking at me?! Some of you do not bath!”)* |
| 48 |  | disclosure of diagnosis to friends/family by HCW |
| 49 |  | no information on illness |
| 49, 38 |  | limited transparency and trust between HCW and patients |
| 46 |  | information by health services didnt include information on reactions, disabilites, med side effects |
| 57, 33 | m | no explanation of illness and treatment by HCW |
| 15, 17 |  | suspicion of coming to clinic |
| 19, 17 |  | long waiting times in clinics (when you go to the clinic you lost that day) |
| 34, 46 |  | delay of diagnosis (2 years) |
| 33 |  | extreme isolation in clinics |
| 17 |  | chronic medicine shortage |
| 46 |  | wrong diagnosis (pneuomonia) |
| 36, 11 | w | no seperation of rooms between f and m, HCW was male, made her feel embarassed |
| 11, 46, 20 |  | wrong diagnosis (more common for women)/only taking her serious when husband told them to (TB was considered to be a disease of men) |
| 2, 45, 27, 20, 53 |  | delivery of diagnosis too harsh, limited information, rude behavior by HCW, scare-mongering by HCW, fear of contagion by HCW |
| 10 |  | delay of diagnosis |
| 45, 20 |  | difficulties with childcare, children not allowed in hospitals, household |
| 45, 6, 20 |  | lack of privacy, crowded hospitals |
| 27 |  | suspicion of coming to clinic |
| 20 |  | corrupt practices from private providers who prescribed vitamins and other supplements instead of TB medicine, paying for free sputum examination |

| ***Positive Experiences*** | | |
| --- | --- | --- |
|  | **Leprosy** |  |
| 7 | m | HCW take sufficient time to talk to patients, physiotherapists teach exercises well |
| 7 | w | no discrimination during treatment |
| 7 |  | HCW are compassionate and considerate |
| 7 |  | priviliged as women during waiting time |
| 18 |  | assistance from beneficiary bodys |
|  | **TB** |  |
| 10, 49, 38, 46 | mw | HCW as kind, dedicated helpful |
| 10 |  | quick start of treatment after diagnosis |
| 17 |  | only trust in government hospital |
| 45 |  | observation through DOTS helped with treatment adherence |
| 45 |  | no gender discrimination from staff |
| 57 | m | nurses even searched for patients in local bars |
| 15 |  | clinics will tell you the truth in comparison to alternative care |
| 2, 6, 20, 46, 53 | w | friendly, informative and supportive HCW (community health agents and nurses particularly close to patients (46) |
| 6 |  | no discrimination during treatment |
| 44 |  | various clinics to seek care from |
| 44 |  | follow up of HCW when patient failed to |

| ***Facing Barriers to Care*** | | |
| --- | --- | --- |
|  | **Leprosy** |  |
| 7 | m | no money for transportation, limited access |
| 29 | w | financial contraints for specific footwar |
|  |  | limited access, dependant on family for transport |
|  | **TB** |  |
| 55, 17, 45, 48, 49, 3, 17, 55 | mw | indirect costs of seeking care (cannot work during that time), transport |
| 10 |  | choose hospitals close to their homes due to transport issues |
| 37, 46 |  | financial constraints to buy recommended food |
| 37 | m | missed work opportunites due to seeking care |
| 3 |  | transport costs |
| 55, 17 |  | indirect financial burden due to treatment |
| 8, 45, 6 | w | low priority of women health |
| 36, 54, 6, 11, 53 |  | transportation difficulties and costs/ not being able to travel alone |
| 43, 44, 53, 20 |  | treatment costs |

*Theme 2*

| **Theme 2: Health Behavior** | | |
| --- | --- | --- |
| ***Making Decisions*** | | |
| **Study ID** | **Gender & Disease** |  |
|  | **Leprosy** |  |
| 7 | w | permission from husband |
| 7 |  | permission from in-laws |
|  | **TB** |  |
| 49 | m | in control of own treatment process *("in my family I am the boss, I decide")* |
| 8, 36, 25 | w | not allowed to go to treatment without male member |
| 32, 36, 50, 3, 11, 50, 32, 45, 49, 6 |  | (no) permission from husband for treatment *(‘No, let her [the mother] die at home" (male)), "women belong home")* |
| 3 |  | unmarried women need no permission |
| 11, 2, 41, 45, 44 |  | (no) permission from mother in law (11,2)/ family (41, 45) mother persisted her daughter went (44) |
| 28 |  | high-decision making power due to good relationship with natal family |
| 41 |  | no permission from family |
| 21 |  | “3 years of not knowing, 3 years after knowing, and 3 years of rotting,” |

| ***Reasons for Care Seeking*** | | |
| --- | --- | --- |
|  | **Leprosy** |  |
| 29, 21 | w | Symptoms worsened |
|  | **TB** |  |
| 10 | mw | staying healthy |
| 10 |  | worried it was serious |
| 10 |  | not being able to work anymore |
| 10 |  | encouragement from loved ones |
| 10 |  | Symptoms worsened |
| 10 | m | lost too much weight |
| 50 |  | risk of diminished marriage prospects |
| 19 |  | encouragement from women |
| 46, 10, 41, 44, 50 | w | symptoms worsened |
| 3, 45, 6, 44 |  | staying healthy for children and family |

| ***Reasons for not seeking care*** | | |
| --- | --- | --- |
|  | **Leprosy** |  |
| 29, 21 | w | Not possible to leave domestic chores/take time off work |
| 29, 21 |  | Symptoms worsened |
| 21 |  | Being neglected in family |
| 50 |  | Afraid to leave parents |
|  | **TB** |  |
| 3, 17, 45 | mw | financial challenges |
| 48 |  | afraid to be recognized at clinic |
| 3, 19 | m | masculinisim *("powerful beings", These men who are like girls are the ones who run to the hospital.” don't want to be labelled weak or not a man)* |
| 10, 3, 17, 15, 33 |  | Symptoms worsened |
| 10, 37, 3, 11, 17, 45, 19 |  | Conflicting work obligations/ economic responsibility (especially harder for those working further away) |
| 50 |  | Isolation |
| 19, 41 |  | Being shy/afraid of other people's perception |
| 34 |  | being an illegal immigrant |
| 8, 41, 44 | w | being a women/ health not prioritized (social norms) |
| 8, 9, 25, 50, 41, 20, 44 |  | no one to accompany her/ unable to travel alone |
| 10, 49 |  | no awareness about disease |
| 11, 45, 41, 44 |  | no access to finances *(In the end, a woman may “regret how much she would spend to know her health.”)* |
| 11, 44 |  | family obligations |
| 11, 44, 25 |  | stigma |
| 20 |  | mistrust in government services |
| 44 |  | fear |

| ***Motivation to Adhere*** | | |
| --- | --- | --- |
|  | **Leprosy** |  |
| 26, 50 | w | wanting to follow HCW instructions |
| 47 |  | marriage prospects |
|  | **TB** |  |
| 10 | mw | desire to get well |
| 46 |  | good relationship with HCW |
| 46 | m | wanting to go back to work |
| 50, 19 |  | support by family |
| 46 |  | married men more likely to adhere |
| 19, 50 |  | maintaining masculine identity associated with fatherhood |
| 19 | w | wanting to protect family |
| 50, 50 |  | determination to get cured |
| 50 |  | alarms/ reminders on phone |
| 1 |  | support from husband/family |
| 9 |  | living to be there for children |
| ***Non-adherence*** | | |
|  | **Leprosy** |  |
| 50 | w | Medication regimen difficult to follow (not on empty stomach) |
|  | **TB** |  |
| 3, 48, 49, 8 | mw | financial challenges/ economic burden |
| 9 | m | intake of medication |
| 32, 37, 50, 8, 19 |  | lost income/ not being able to work |
| 37, 19 |  | no money for food |
| 9, 50 |  | interference with alcohol/ drug addiction |
| 49 |  | disbelief in healthcare |
| 46 |  | single men more likely to forget daily medication |
| 14 |  | discomfort of looking weak to community |
| 6, 44 | w | lack of funds/ dependant on husband |
| 6, 20 |  | side effects |
| 6, 46 |  | psychological barriers |
| 6, 32, 50, 49 |  | family responsibilities *"I took the drugs for about 6 months and then left, being a defaulter... What they say is: “we need someone who will serve us...” (32)* |

*Theme 3*

| **Theme 3: Livelihood Effects** | | |
| --- | --- | --- |
| ***Changes in Daily Life*** | | |
| **Study ID** | **Gender & Disease** |  |
|  | **Leprosy** |  |
| 7 | m | resting from household chores |
| 16 |  | still attended social life |
| 18 | w | stopped with self-care due to appearance change |
| 7, 21, 47 |  | resuming household chores (even with pain) |
| 7, 12, 47, 51, 52 |  | lack of sleep, sleeping seperately, loss of intimicy with loved ones |
| 26 |  | continued with life as usual |
| 29, 21, 47 |  | impact on mobility, daily tasks due to nerve pain |
| 12, 24, 40, 47, 52, 30, 7 |  | avoids social activities, outside, isolation |
| 23 |  | missed opportunities due to fear of spreading disease |
|  | **TB** |  |
| 4 | mw | avoid social activities, outside, isolation |
| 31, 4, 15 | m | no food/ starvation/weightloss (unmarried men have no one to cook for them) |
| 4, 48, 15, 34 |  | avoid social activities, outside, isolation |
| 4, 15 |  | physical strengths/ sexual performance affected |
| 50 |  | kept working when self-employed |
| 50, 45 | w | losing work |
| 1, 9, 37, 25, 41, 5, 53 |  | difficulty with household chores (role of wife is to take care of husband, children, household) |
| 1, 50, 57, 6, 20, 44 |  | continue with chores, (pressure) to appear healthy |
| 57, 4, 25, 49, 5, 6, 27, 53, 8, |  | avoid social activities, outside, isolation |
| 25, 5, 27 |  | concerns over potential risks of TB during pregnancy |
| 25, 47 |  | denied to see baby |
| 5 |  | infertility, unfullfilled wish to become parents |

|  |  | ***Work & Finances*** |
| --- | --- | --- |
|  | **Leprosy** |  |
| 7 | m | rest from employment (no discrimination) |
| 7 |  | loss of income and promotions due to frequent leaves |
| 16 |  | inability to go to work due to symptoms, living on wifes income |
| 18 | w | domestic work affected |
| 18, 23, 42 |  | loss of employment (also due to disability) |
| 47, 30 |  | change of employment (One woman who lived in the community even recycled newspapers or magazines to exchange for cash) |
|  | **TB** |  |
| 50, 3, 48 | mw | decrease of wages, loss of income |
| 32, 1, 37, 11, 48, 31, 19 | m | pressure of providing for family in a context where men are financial pillars (men that they must appear strong, and men mentioned disregarding TB symptoms: *“Men think they are strong, the woman is waiting for you to bring money, you don’t have time to think, you have to work”)* |
| 57, 37, 11, 48, 57, 19, 8 |  | leaving/loosing job due to pain/symptoms/ resigning voluntary to not be rejected |
| 3, 11, 31 |  | (expected to/ have to) work despite illness |
|  |  | *(19) Not only does loss of employment result in financial insecurity and food purchasing power but also a direct pathway between employment and food security emerges when food is the currency of payment for work. Furthermore, while some men were able to access food resources when unemployed, the increased hunger induced by TB treatment increased the need for and cost of food* |
|  |  | *(19)co-workers noticed his symptoms and complained about his coughing to the boss. When confronted, he explained that he would go to the clinic, presumably to get tested and initiate treatment. However, in the process of acknowledging his illness, he was fired to prevent other employees from getting TB and lowering work performance.* |
| 31 | w | exempted from house chores |
| 13, 20, 44, 53 |  | difficulty to work/ inability to work economic insecurity |
| 57 |  | continuing with work (no other option) |
| 4, 2 |  | seperated from others at work/ school |
| 49 |  | *Did you get any wages from your employer during your sick leave? Respondent: I got 2 1/2 months’ wages, but each employee at the company where I work contributed one day of their wages each.... Thirty people contributed 300,000 Dong totally. They brought the money to me when I was in hospital.* |
| 2 |  | dependancy on husband |

| ***Concealment and Disclosure*** | | |
| --- | --- | --- |
|  | **Leprosy** |  |
| 16 | m | disease only discussed with wife or doctor |
| 26, 12, 23, 24 | w | non-disclosure because of people's prejudice |
| 26, 12, 42 |  | concealment through long clothes/ closed sneakers |
| 26 |  | avoiding conversations about health |
| 26, 23, 35 |  | hiding diagnosis from family and friends |
| 12, 24, 35 |  | only disclosed diagnosis to husband and children |
| 12, 21, 24, 47, 52 |  | self-isolation |
| 12, 47, 56 |  | telling people they have a less stigmatized disease (e.g. skin cancer, allergies) |
| 24, 12 |  | not telling husband fearing divorce |
| 40 |  | concealment due to feaing rejection |
|  | **TB** |  |
| 25, 45 | m | no issue with disclosure |
| 9, 57, 4, 38, 34, 33 |  | isolation/ avoidance |
| 9, 14, 15 |  | disclosure only to select persons |
| 57 |  | leaving work voluntary to not disclose dignosis |
| 57, 49, 15, 33, 9 |  | concealment fearing rejection, *job loss* |
| 33 |  | concealment because of link to HIV |
| 25, 27 | w | terrified HCW may disclose diagnosis to family when visting her home |
| 37 |  | concealment to protect family reputation |
| 50, 55, 11, 25, 41, 2, 45, 6, 27, 28, 43, 53 |  | concealing (to escape social consequences) |
| 4, 53 |  | disclosure only to select persons |
| 41, 48, 54, 27, 28, 44 |  | not telling husband fearing divorce/marriage prospects |
| 48, 2, 44, 53, 22 |  | isolation/ avoidance |
| 43 |  | concealment because of link to HIV |
|  |  | *(43) “[If they know you have TB] they start treating you like you’re not a human being like them.”* |

*Theme 4*

| **Theme 4: Psychological Factors** | | |
| --- | --- | --- |
| ***Mental Health*** | | |
| **Study ID** | **Gender & Disease** |  |
|  | **Leprosy** |  |
| 7 | m | anxiety, depression, suicidal thoughts, feeling of segregation |
| 50, 21 | w | changing of skin/ appearance |
| 7, 26, 12, 21, 24, 40, 50 |  | depression, sadness |
| 7, 26, 21 |  | fear |
| 26, 29, 12, 21 |  | frustration, distress |
| 26, 21, 24 |  | anger |
| 21, 24, 40, 47 |  | self-harm, suicidal thoughts |
| 21, 24 |  | acceptance, self-confidence |
|  |  | *(47)Women in this study mentioned that, even though they were physically free of the microorganisms, they would never be free of the leprosy emotionally* |
|  | **TB** |  |
| 10, 49 | mw | depression, fear |
| 4 | m | loneliness |
| 46 |  | appearance |
| 46, 34, 33, 5, 6, 50 |  | sadness, depression |
| 34, 5, 6, 27, 50 |  | suicidal thoughts |
| 1, 13, 2, 27, (38) | w | depression, sadness |
| 13, 50 |  | helpless |
| 48, 5, 27, 50 |  | fearful, frightened |
| 2, 1, 50, 5 |  | suicidal thoughts, self-harm(2 ‘*I don’t want to live anymore... I want to commit suicide. Tomorrow, either I will leave this hospital, or my body will be taken out."*) |
| 5 |  | emotional break down |
|  |  | general reasons: because of rejection, not being able to care for family, fear of progression, suicide often stopped because of not wanting to burden family further |

| ***(Distorted) Self-Image (masculinity, femininity)*** | | |
| --- | --- | --- |
|  | **Leprosy** |  |
| 26, 12, 21,23, 24, 40, 50, 50, 50 | w | stigma and blame projected onto herself (guilt, insecure, inferior, ashamed, embarrassed) |
| 23, 50, 42, 18, 21, 50, 50, 30, 29, 40, 47 |  | impaired self-image due to physical changes *(42, Expressions like “rotten”, “leprous”, “frog leather”, “snake skin” and “rotten bean” are pejorative terms some participants used to name// I look like a monster // I do not find myself a complete woman because of the disease. According to me, a real woman is a healthy woman, in good health. I differ from the others. I am a woman, but I do not longer feel like a whole woman, a woman like I was before)* |
| 47, 23, 47 |  | not as competent as other mothers due to disease (physical touch affected) |
|  |  | *(47) It’s like there is a mark on your forehead that says you are a leprosy patient. Even now, I still call myself a patient and people without leprosy healthy persons.* |
|  |  | *(50) ""Why would they take us if our hands  were wasted? They have married our hands which cook and clean and sweep!* |
|  | **TB** |  |
| 4, 37, 11 | m | feeling embarrassed, shy, ashamed |
| 4, 14, 19, 34, 37, 25 |  | not feeling like a man (being weak, not able to provide, loosing control) (19 *Through the process of enduring pain and recovering from illness, men prove their strength and manliness.*) |
| 45, 4 | w | impaired self-image due to physical changes (excessive weightloss) |
| 4, 38, 2, 27, 44, 50, 46 |  | stigma and blame projected onto herself (guilt, insecure, inferior, ashamed, embarrassed) |

| ***(No) Worries and Concerns*** | | |
| --- | --- | --- |
|  | **Leprosy** |  |
| 16 | m | afraid of disabilities causing marital problems or unemployment |
| 26, 30 | w | no worries about diagnosis |
| 29, 21, 40, 47 |  | reactions, disabilities, physical changes |
| 12, 40 |  | infecting family and others |
| 18 |  | financial stabiliy |
| 23, 47, 24, 40 |  | marriage prospects/problems/ rejection |
| 24 |  | people finding out |
|  | **TB** |  |
| 13, 22, 49, 48, 45 | m | no worries/ limited worries |
| 50, 38, 48, 49 |  | loss of wages/ employment |
| 50 |  | fear of other men taking over household |
| 4, 38, 14 |  | fear of comments/ rejection |
| 38 |  | death |
| 38, 19 |  | infecting family and others |
| 38, 50, 4, 11, 49, 46, 2, 45, 41, 6, 27, 25, 1 | w | rejection from husband/community |
| 13, 9, 48, 1 |  | inability to take care of children/family |
| 50, 38, 28, 44 |  | dismissal from work |
| 48, 2, 38 |  | infecting family and others |
| 38 |  | death, 50 |
| 43 |  | association with HIV |

*Theme 5*

| **Theme 5: Effects on Relationships** | | |
| --- | --- | --- |
| ***Community*** | | |
| **Study ID** | **Gender & Disease** |  |
|  | **Leprosy** |  |
| 26, 23, 24, 40, 35, 52, 56, 42, 21, 47, 52 | w | social stigma: avoidance/ gossip/judgement from colleagues/ friends |
| 12, 24, 7 |  | no social stigma |
| 16 | m | no social stigma |
|  | **TB** |  |
| 13, 45, 31, 7, 37 | m | no social stigma |
| 4, 48, 14, 34, 33, 15 |  | social stigma: avoidance/ gossip/ judgement from colleagues/ friends; (36) ‘*The social effects sometimes are worse than the illness itself [...] // (4) Judgment towards men often arose from behaviors perceived to be related to contracting TB* (e.g., drinking alcohol, smok-ing, partying). |
|  |  | *(45) men were also more commonly not faced by the opionion of others contrasting to women* |
|  |  | *(34) forced away from community* |
| 13, 22, 37, 57, 4, 48, 45, 6, 27, 43, 44, 53, 1, 50, 46, 2, 27 | w | social stigma |
|  |  | *(37) judged as lazy by neighbors due to TB and effects on daily life* |
|  |  | *facing eviction, verbal abuse, avoidance due to fear of contagion, tease you, disgusted of them* |
|  |  | *(43) “[If they know you have TB] they start treating you like you’re not a human being like them.”* |

| ***Family and In-Laws*** | | |
| --- | --- | --- |
|  | **Leprosy** |  |
| 7, 35 | w | neglect from in-laws |
| 23, 47, 51 |  | avoidance from family |
| 47 |  | no change in treatment |
| 52 |  | hidden by family |
| 47 |  | *(47) relative washed the chair that she had sat on for just a few minutes. Another woman told a story about her older brother’s fear of touching her hands.* |
| 51 |  | *(51) One woman said she had  been served food in a dog's dish* |
|  | **TB** |  |
| 55, 22, 49 | m | positive attitude of family towards disease |
| 4, 41, 48, 14, 19 |  | hostility, exclusion, isolation from family |
| 4 |  | *(4) "being locked in their rooms by family due to perceived safety concersns"* |
| 55, 22, 7, 1, 8, 13, 22, 57, 45, 41, 27, 20, 53 | w | hostility, exclusion, isolation, discrimination from family |
| 50, 2, 27, 28, 20 |  | rejection, mistreatment by mother-in-law |
| 2, 20 |  | avoidance from in laws |
| 2, 28, 43 |  | support from own family |
| 20 |  | *(20) woman’s nutritional needs often come last within the family. Furthermore, discriminatory practices against female TB patients could mean denial of food from family members.* |
| 28 |  | *(28) In Parul’s case, her illness seems to be used as an excuse by her husband and in-laws to get rid of a problematic wife.* |

*Theme 6*

| **Theme 6: Coping** | | |
| --- | --- | --- |
| ***Recommendations*** | | |
| **Study ID** | **Gender** |  |
|  | **TB** |  |
| 17 | m | provide/stiffen employment laws subjecting supervisors to maltreatment |
|  |  | all workers should be on medical scheme |
|  |  | educationally oriented interventions should foster gradual reforms, behavior change, public awareness and civic education programs |
|  |  | establising own social safety nets, e.g. village fund where everyone contributes a little bit, so people can go to hospital by ambulance |
|  |  | outreach service, see people and esp. men in their homes to take away from financial barriers |
|  |  | setting up feedback options at hospitals to forward complaints against clinical staff to stop il treatment |
|  | mw | framing of researchers that traditional medicine is wrong and people would be punished was not liked by community |
|  |  | refund transport costs to clinic to encourage care-seeking |
| 45 | w | outreach service, HCW come to their home to ease difficulties with childcate (in health posts children werent allowed to come in) |
| 20 | w | food incentives from governemt to improve nutrition for women once a month (important for medication intake) |
| 44 | w | outreach service, to more economically depresed areas |
|  |  | improve efficiency, interactions and timeliness at health facilities to improve care-seeking |
| 14 | m | spread information for men at common social spots & comfort spots, start at taverns where cigarettes and beers are shared |
|  |  | large-scale community and family-centered TB awareness-raising events, promoted through text messaging and loudspeaker announcements |
|  |  | desire and willingness to support men, as they fear to go to clinic, accompany them, help with sputum collection |
| 10 | mw | case finding in bars and taverns could help better reach and diagnose TB among men, as they spend a lot of time there |
|  |  | women on the other hand were said to be ‘into church’ and approaching them at churches would help in reaching them and detecting TB sooner |

| ***Coping Strategies*** | | |
| --- | --- | --- |
|  | **TB** |  |
| 4 | mw | family networks providing resources to combad loneliness and sustain resilience, empathy from friends, reastablishing of conncetions associated with improves health and strenghts |
| 32 | w | families received counselling, living seperately from in-laws |
| 2 | w | maintaining economic independence through online work, reading books, watching movies for distraction |
|  |  | religion |
|  |  | peer-to-peer support through shared experiences (provided joy and relief) |
|  |  | key coping mechanism was mental support from family and spouse |
| 27 | w | peer-to-peer support at clinics |
|  |  | religion |
|  |  | listening to music, watching TV |
|  |  | focusing on school to mitigate stress of dealing with TB, made them feel positive |
|  |  | accepting illness as destinity in a fatalistic way |
| 53 | w | willpower |
| 14 | m | peer-to-peer support |
|  | **Leprosy** |  |
| 21 | w | religion |
| 23 |  | religion, God's destiny |
| 40 |  | inner acceptance and peace |
|  |  | religion, spirituality |
| 47 |  | religion |
|  |  | religion |
| 52 |  | religion |
|  |  | children as meaning of life |
| 56 |  | religion, a higher power helps "only God can give us strengths" |
|  |  | willpower |
| 30 |  | willpower |
|  |  | social role of being a mother as important coping factor "carry on with things" |
| 42 |  | religion |
|  |  | other people are worse off "I'm fine compared to that person, right?" |
|  |  | willpower |
|  |  | self-care management for the bodily skin (oils, creams etc.) |

| ***Support (from)*** | |
| --- | --- |
| m | spouses (also ex) |
|  | family support (also children) |
|  | sisters |
|  | brothers distanced themselves |
|  | mothers in law |
|  | community, friends |
|  | ‘to be honest brother, [...] the moral support is more important than the financial support’ |
| w | family support (also children) |
|  | in-laws |
|  | spouses |
|  | mother |
|  | brother |
|  | grandmother |
|  | no support/little support |
|  | harder for unmarried women |
|  | HCW & doctors |
|  | community: neighbors, friends |
|  | social worker |
|  | Married women patients, however, did not experience the same degree of family support as did unmarried women |
|  | womens support networks were smaller than men |
| mw | Gender and marital status appeared to be key criteria in respondents' varied perception of level of family support. |
