# Supplementary material for: Gendered experiences of people living with tuberculosis or leprosy: A global systematic review
Source: PLoS Negl Trop Dis. 2026 Sep 8;20(9):e0014711. doi: 10.1371/journal.pntd.0014711 (PMC13577493; doi:10.1371/journal.pntd.0014711)
Supplement: Supplementary file 5 [file pntd.0014711.s005.docx]

**S4 File**

**Quality Appraisal of Included Studies**

| **Qualitative Research CASP Checklist** | | | | | | | | | | | |
| --- | --- | --- | --- | --- | --- | --- | --- | --- | --- | --- | --- |
| Legend: Y= Yes, N=No, ?=not mentioned, *=comment | | | | | | | | | | | |
| **Study** | **Section A: Are the results valid?** | | | | | | **Section B: What are the results?** | | | **Section C:**  **Will results help locally?*** | |
|  | Was there a clear statement of the aims of the research? | Is a qualitative methodology appropriate? | Was the research design appropriate to address the aims of the research? | Was the recruitment strategy appropriate to the aims of the research? | Was the data collected in a way that addressed the issue? | Has the relationship between researcher and participant been adequately considered? | Have ethical issues been taken into consideration? | Was the data analysis sufficiently rigorous? | Is there a clear statement of findings? | *Are limitations/ biases addressed? | Comments |
| **Qualitative Studies** | | | | | | | | | | | |
| Stein et al. (2025) | Y (p.2) | Y (p.2-3) | Y (p.2-3) | Y (p.3) | Y (p.3) | Y (p.3,8) | Y (p.4) | Y (p.4ff) | Y (p.9) | Y (p.8) |  |
| Brüggemann et al. (2025) | Y (p.3) | Y (p.3) | Y (p.3) | Y (p.4) | Y (p.4-5) | Y (p.12) | Y (p.5) | Y (p.4) | Y (p.13) | Y (p.12) |  |
| Muttamba et al. (2024) | Y (p.2) | Y (p.2) | Y (p.2) | Y (p.3) | Y (p.3) | Y (p.3) | Y (p.4) | Y (p.3) | Y (p.8) | Y (p.8) |  |
| Medina-Marino et al. (2025) | Y (p.2) | Y(p.3-4) | Y (p.4) | Y (p.3-4) | Y (p.4) | Y (P.4) | Y (p.14) | Y (p.5) | Y(p.13) | Y (p.13) |  |
| Akther et al. (2024) | Y (p.2) | Y (p.2) | Y (p.2-3) | Y (p.2) | Y (p.2-3) | ? | Y (p.7-8) | Y (p.3) | Y (p.7) | Y (p.3,7) |  |
| Guspianto et al. (2024) | Y (p.428) | Y (p.428) | Y (p.428) | ? | Y (p.428) | ? | Y (p.428) | ? | Y (p.432) | Y (p.432) |  |
| Sharma et al. (2023) | Y (p.44) | Y (p.44-45) | Y (p.44-45) | Y (p.45-46) | Y (p.45) | ? | Y (p.45) | Y (p.45) | Y (p.55) | N |  |
| Abubakar et al. (2023) | Y (p.2) | Y (p.2) | Y (p.2) | Y (p.2) | Y (p.2) | Y (p.2) | Y (p.6) | Y (p.2) | Y (p.6) | Y (p.6) |  |
| Turusbekova et al. (2022) | Y (p.2) | Y (p.2-3) | Y (p.2-3) | Y (p.2-3) | Y (p.2-3) | Y (p.8) | Y (p.3) | ? | Y (p.8) | Y (p.8) |  |
| Susanto et al. (2023) | Y (p.21) | Y (p.21) | Y (p.21) | Y (p.21-22) | Y (p.22) | Y (p.22) | Y (p.23) | Y (p.22-23) | Y (p.33) | Y (p.32) |  |
| Huq et al. (2022) | Y (p.2) | Y (p.3) | Y (p.3) | Y (p.3) | Y (p.3) | Y (p.3) | Y (p.17) | Y (p.5) | Y (p.17) | Y (p.16) |  |
| Medina-Marino et al. (2022) | Y (p.3) | Y (p.3) | Y (p.3) | Y (p.3) | Y (p.3) | Y (p.3) | Y (p.4) | Y (p.3-4) | ?* | N | *? Results, discussions yes, but no seperate conclusions |
| Makgopa et al. (2022) | Y (p.2) | Y (p.2-3) | Y (p.2-3) | Y (p.2-3) | Y (p.3) | ? | Y (p.4) | Y (p.4) | Y (p.11) | N* | N* addressed only in one sentence, not sufficient enough |
| Meis et al. (2022) | Y (p.71) | Y (p.71) | Y (p.71) | Y (p.72) | Y (p.73) | Y (p.73) | Y (p.74) | Y (p.74) | Y (p.85) | Y (p.84) |  |
| Phiri et al. (2021) | Y (p.2) | Y (p.2-3) | Y (p.2-3) | Y (p.3) | Y (p.3) | Y (p.3) | Y (P.9) | Y (p.3) | Y (p.8) | Y (p.8) |  |
| Goncalves et al. (2021) | Y (p.2) | Y (p.2) | Y (p.2) | Y (p.3) | Y (p.3) | Y (p.3) | Y (p.3) | Y (p.3) | Y (P.9) | Y (P.9) |  |
| Daniels et al. (2021) | Y (p.2) | Y (p.2) | Y (p.2) | Y (p.2) | Y (p.2) | ? | ? | Y (p.2) | Y (p.6) | Y (p.5) |  |
| Mukerji & Turan (2020) | Y (p.1719) | Y (p.1719-20) | Y (p.1719-20) | ? | Y (p.1719-20) | ? | Y (p.1720) | Y (p.1720) | ?* | Y (p.1727) | *? Results, discussions yes, but no seperate conclusions |
| Jung & Yang (2020) | Y (p.2) | Y (p.2-3) | Y (p.2-3) | Y (p.3) | Y (p.3) | ? | Y (p.3) | Y (p.3) | Y (P.9) | N* |  |
| Nasir et al. (2020) | Y (p.306) | Y (p.306-7) | Y (p.306-7) | Y (p.306-7) | Y (p.307) | ? | ? | Y (p.307) | Y (p.310) | N |  |
| Kumar et al. (2020) | Y (p.65) | Y (p.66) | Y (p.66) | ? | ? | Y (p.66) | Y (p.66) | ? | Y (p.75) | N |  |
| Hatherall et al. (2019) | Y (p.1110) | Y (p.1110) | Y (p.1110) | Y (p.1110) | Y (p.1110-11) | Y (p.1110-11) | Y (p.1110) | Y (p.1111) | ?* | Y (p.1117) | *? Results, discussions yes, but no seperate conclusions |
| Neiva & Grisotti (2018) | Y (p.3) | Y (p.3) | Y (p.3) | ? | Y (p.3) | ? | Y (p.3) | ? | Y (p.16) | N |  |
| Mukerji & Turan (2018) | Y (p.728) | Y (p.728) | Y (p.728) | Y (p.728) | Y (p.728) | ? | Y (p.729) | Y (p.728) | ?* | Y (p.733) | *? Results, discussions yes, but no seperate conclusions |
| Barua et al. (2018) | Y (p.4) | Y (p.5) | Y (p.5) | Y (p.5) | Y (p.5) | ? | Y (p.7) | Y (p.5-6) | Y (p.16) | N |  |
| Silva & Barsaglini (2018) | Y (p.3) | Y (p.4) | Y (p.4) | ? | Y (p.4) | ? | Y (p.4) | Y (p.4) | Y (p.14) | N |  |
| Goncalves et al. (2017) | Y (p.662) | Y (p.662) | Y (p.662) | Y (p.662) | Y (p.662) | ? | Y (p.662) | Y (p.662) | Y (p.666) | Y (p.666) |  |
| Miller et al. (2017) | Y (p.43) | Y (p.43) | Y (p.43) | Y (p.43) | Y (p.43) | Y (p.43) | Y (p.43) | Y (p.43) | Y (p.47) | Y (p.47) |  |
| Horter et al. (2016) | Y (p.2) | Y (p.2-3) | Y (p.2-3) | Y (p.2-3) | Y (p.3) | Y (p.12-13) | Y (p.14) | Y (p.3) | Y (p.13) | Y (p.12-13) |  |
| Chinouya & Adeyanju (2017) | Y (p.46) | Y (p.46) | Y (p.46) | Y (p.46) | Y (p.46) | Y (p.46) | Y (p.46) | Y (p.46) | Y (p.49) | Y (p.49) |  |
| Zuniga et al. (2016) | Y (p.33) | Y (p.33-34) | Y (p.33-34) | Y (p.33-34) | Y (p.33-34) | ? | Y (p.33) | Y (p.34) | Y (p.36-37) | Y (p.36) |  |
| van't Noordende et al. (2016) | Y (p.2) | Y (p.2-3) | Y (p.2-3) | Y (p.2-3) | Y (p.3) | Y (p.3) | Y (p.3) | Y (p.3) | Y (p.7) | Y (p.7) |  |
| Shiotani & Hennik (2014) | Y (p.1240) | Y (p.1241-42) | Y (p.1241-42) | Y (p.1241-42) | Y (p.1241-42) | ? | Y (p.1242) | Y (p.1242) | Y (p.1250) | N |  |
| Velez & Montoya (2015) | Y (p.219) | Y (p.219-20) | Y (p.219-20) | Y (p.219-20) | Y (p.219-20) | ? | Y (p.220) | Y (p.219-20) | ?* | Y (p.224) | *? Results, discussions yes, but no seperate conclusions |
| Chikovore et al. (2014) | Y (p.2) | Y (p.3) | Y (p.3) | Y (p.3) | Y (p.3) | Y (p.4) | Y (p.4) | Y (p.4) | Y (p.10-11) | Y (p.11) |  |
| Peters et al. (2014) | Y (p.7) | Y (p.8-9) | Y (p.8-9) | Y (p.8-9) | Y (p.8-9) | ? | Y (p.9) | ?* | Y (p.18-19) | Y (p.18) | *? Data analysis mentioned only briefly |
| Khan (2011) | Y (p.5) | Y (p.6-7) | Y (p.6-7) | Y (p.7) | Y (p.6-7) | Y (p.7) | ? | Y (p.7) | Y (p.14-15) | N |  |
| Palmeira & Ferreira (2012) | Y (p.380) | Y (p.380-81) | Y (p.380-81) | Y (p.381) | Y (p.380-81) | ? | Y (p.381) | Y (p.381) | Y (p,385) | N |  |
| Macfarlane & Newell (2011) | Y (p.143) | Y (p.144) | Y (p.144) | Y (p.144) | Y (p.144) | Y (p.146) | Y (p.147) | ?* | ?* | Y (p.146) | *? Data analysis mentioned only briefly in one sentence; *? Results, discussions yes, but no seperate conclusions |
| Hu et al. (2011) | Y (p.30) | Y (p.31-33) | Y (p.31-33) | Y (p.32) | Y (p.32) | ? | Y (p.32) | Y (p.33) | Y (p.43) | Y (p.43) |  |
| Onifade et al. (2010) | Y (p.2) | Y (p.2-3) | Y (p.2-3) | Y (p.2) | Y (p.3) | Y (p.10) | Y (p.3) | Y (p.3) | Y (p.10) | Y (p.10) |  |
| Nogueira & Queiroz (2010) | Y (p.628) | Y (p.628-29) | Y (p.628-29) | Y (p.629) | ?* | ? | Y (p.629) | ?* | Y (p.634) | ?* | *? Data collection, data analysis and limitations only briefly mentioned in one sentence |
| Shieh et al. (2006) | Y (p.100-1) | Y (p.101-4) | Y (p.101-4) | Y (p.101-2) | Y (p.101-4) | ? | Y (p.104) | Y (p.104) | Y (p.112) | N |  |
| Long et al. (2001) | Y (p.70) | Y (p.70-72) | Y (p.70-72) | Y (p.71) | Y (p.70-72) | ? | Y (p.72) | ?* | ?* | N | *? Data analysis mentioned only briefly in one sentence; *? Results, discussions yes, but no seperate conclusions |
| Johansson & Wikvist (2002) | Y (p.475) | Y (p.475-77) | Y (p.475-77) | Y (p.475) | Y (p.475-76) | Y (p.476) | Y (p.476) | Y (p.477) | Y (p.489) | N |  |
| Khan et al. (2000) | Y (p.248) | Y (p.248-49) | Y (p.248-49) | Y (p.248-49) | Y (p.248-49) | ? | ? | ?* | Y (p.254) | N | *? Data analysis mentioned only briefly in one sentence |
| Oliveira et al. (1999) | Y (p.86) | Y (p.86) | Y (p.86) | Y (p.87) | Y (p.86-87) | ? | ? | Y (p.86-87) | Y (p.89) | N |  |
| Nair et al. (1997) | Y (p.78-79) | Y (p.79) | Y (p.79) | Y (p.79) | Y (p.79) | ? | ? | ? | Y (p.84) | Y (p.79) |  |
| Liefooghe et al. (1995) | Y (p.1686) | Y (p.1687) | Y (p.1687) | ? | Y (p.1687) | Y (p.1687) | ? | Y (p.1687) | Y (p.1691) | Y (p.1691) |  |
| Palmeira et al. (2013) | Y (p.894) | Y (p.894-5) | Y (p.894-5) | Y (p.894-5) | Y (p.894-5) | ? | Y (p.895) | Y (p.895) | Y (p.898) | N |  |
| Jung et al. (2020) | Y (p.48) | Y (p.48-49) | Y (p.48-49) | Y (p.49) | Y (p.48-49) | Y (p.49) | Y (p.49) | Y (p.49) | Y (p.54) | N |  |
| Omona et al. (2024) | Y (p.3) | Y (p.4-5) | Y (p.4-5) | Y (p.4-5) | Y (p.5) | ? | Y (p.5) | Y (p.5) | Y (p.21) | N |  |
| **Mixed-Method-Studies** | | | | | | | | | | | |
| Vlassoff et al. (2015) | Y (p.120) | Y (p.120) | Y (p.120) | ? | Y (p.120) | ? | ? | ? | Y (p.124) | N |  |
| Chandru & Varma (2023) | Y (p.2) | Y (p.2) | Y (p.2) | Y (p.2) | Y (p.2) | Y (p.3) | Y (p.4) | Y (p.3) | Y (p.12) | Y (p.12) |  |
| Kerkhoff et al. (2023) | Y (p.2-3) | Y (p.3-6) | Y (p.3-6) | Y (p.4) | Y (p.4-5) | Y (p.6) | Y (p.4) | Y (p.5) | Y (p.20) | Y (p.19) |  |
| Mbuthia et al. (2020) | Y (p.2-3) | Y (p.3-5) | Y (p.3-5) | Y (p.3) | Y (p.3-5) | Y (p.4) | Y (p.5) | Y (p.4) | Y (p.11) | ?* | *Limitations addressed but only briefly |
| Anderson de Cuevas et al. (2014) | Y (p.2) | Y (p.2) | Y (p.2) | Y (p.2) | Y (p.2) | ? | Y (p.2) | Y (p.2) | Y (p.8) | Y (p.7) |  |

* The question: "How valuable is the research?" from Section C was answered for all studies with "Yes". It was removed from the table to improve readibility.

* The question “Are limitations/ biases addressed?” was introduced by SCWU, to address a gap in the original checklist and strengthen quality appraisal.
