# Supplementary material for: Gendered experiences of people living with tuberculosis or leprosy: A global systematic review
Source: PLoS Negl Trop Dis. 2026 Sep 8;20(9):e0014711. doi: 10.1371/journal.pntd.0014711 (PMC13577493; doi:10.1371/journal.pntd.0014711)
Supplement: Supplementary file 6 [file pntd.0014711.s006.docx]

**S5 File**

**Coding Framework**

| **Code** | **Definition** | **Anchor example** |
| --- | --- | --- |
| **1. Quality of Care** | | |
|  | Positive and negative examples of care experiences are described, (e.g. including healthcare worker (HCW)-patient relationship, diagnostic accuracy, care settings, privacy aspects and availability of medicines). Results differentiate between men and women. | *“At the first visit the doctor did not refer me to check for tuberculosis, saying there was a low probability, considering I was a woman and probably had not engaged in TB risk behavior.” (WATB, Georgia)*  *“I feel great. The treatment and care are great [...] the care at this facility, for me, is top notch.” (MATB, Brazil)* |
| **2. Health Behavior** | | |
| 2.1. Gendered decision-making power and care-seeking | Decision-making differences about care between men and women are outlined, along with reasons to seek care (e.g. underlying motivations) and reasons not to seek care (e.g. socio-economic barriers). | *“We have such a mentality that a woman in the family does not have a voice, she is sick, but she cannot go to a medical institution, because of housework, child-care, or you need to get permission from your mother-in-law, demand money from your husband, considering all this, a woman does not go to a medical facility until she falls down.” (WAL, Tajikistan)*  *“[…] These men who are like girls are the ones who run to the hospitals.” (MATB, Uganda)* |
| 2.2. Treatment Adherence | Motivations for treatment adherence and barriers to adherence are described. Results differentiate between men and women. | *“[…] I took the drugs for about 6 months and then left, being a defaulter…What they [family] say is: we need someone who will serve us.” (WATB, Uzbekistan).*  *“I couldn’t afford these foods, so I stopped the medicines.” (MATB, India)* |
| **3. Livelihood Effects** | | |
| 3.1. Adjustment to Daily Life During Illness | The impact of TB or leprosy on daily activities are outlined, distinguishing between changes experienced by women and men, including socio-economic effects. | *“I want to work outside, but it is too much of a hassle to go outside, so I stay here doing the laundry and feeding the pigs.” (WAL, South Korea)*  *“I had to stop a lot of things, and I was not able to go to places that I used to go [to]. Places I used to go and smoke at.” (MATB, South Africa)* |
| 3.2. Work & Finances | The impact of TB or leprosy on work and financial status is displayed. This includes differences between men and women in how income and employment are affected and the influence of social role expectations on work responsibilities. | *“I was forced to leave my job, because I was suffering from this disease and I only concentrated on healing the disease.” (WAL, Indonesia)*  *“[…] In the process of acknowledging his illness, he was fired to prevent other employees from getting TB and lowering work performances.” (MATB, South Africa).* |
| 3.3. Concealment and Disclosure | Captures disclosure decisions (concealment vs. disclosure) among men and women, including underlying reasons, consequences and strategies to conceal the disease. | *“The disease was also on my face. I did not feel very comfortable. Therefore, I never socialized with the neighbors … I always wear long sleeves. Today I wear short sleeves so you can see my condition, so you can see and understand my condition. Normally, I use long sleeves. I do it because the neighbors won’t know, because if they know, they will always ask what has happened to me.” (WAL, Indonesia)*  *“I keep the tablets inside this (a blue pouch) ... so that I don’t have to show it to anyone... No one in my neighborhood knows about this.” (MATB, India)* |
| **4. Psychological Effects** | | |
| 4.1. Mental Health | The mental health struggles associated with being affected by leprosy or TB are described, including common fears and concerns, differentiated by men and women. | *“[…] Even though [we are] physically free of the microorganisms, [we] would never be free of the leprosy emotionally.” (WAL, Taiwan).*  *“[…] I was desperate and I even thought about killing myself because of this illness.” (MATB, UK)* |
| 4.2. (Distorted) Self-image | Impact of leprosy or TB on men and women’s self-esteem and self-image are described, including their perceptions on how the diseases affect their femininity or masculinity. | *“I do not find myself a complete woman because of the disease. “[…] A real woman is a healthy woman, I differ from others […].” (WAL, Brazil).*  *I…[interacting] with men I don’t know what to say because I don’t even feel like a man anymore. I feel too weak to be a man because I can’t even provide for myself. How would a man like me provide for a woman? That’s why I don’t even have a girlfriend anymore because I know that I can’t even provide for myself.” (MATB, South Africa)* |
| **5. Effects on Relationships** | | |
| 5.1. Community | Reactions from the community to the participant’s disease status are shared, including stigma, discrimination and changes in social behaviour, differentiated by men and women. | *“If they know you have TB they start treating you like you’re not a human being like them.” (WATB, Uganda).*  *“The social effects sometimes are worse than the illness itself […].” (MATB, Yemen)* |
| 5.2. Marriage and Partnerships | The impact of leprosy or TB on marriage/ marriage prospects and partnerships is outlined, differentiated by men and women. | *“When my husband heard about the disease, he immediately sent me back home, saying he did not want a sick and useless wife with doubtful loyalty.” (WATB, India).*  *“No, I wasn’t treated differently. In fact, my wife helped me a lot. She helped me to follow the doctor’s advice and there was a gradual improvement in me.” (MAL, Nepal)* |
| 5.3. Family and In-Laws | Reactions and behavior change from family and in-laws toward people affected by TB or leprosy are described, differentiating between men and women. | *“My mother-in-law had told me to divorce her daughter and she told her daughter that she would arrange another boy to marry her.” (MAL, Nepal)*  *“One day, the whole family members have invited in the marriage ceremony of my nephew (my brother son), but my own brother didn’t allow me to attend the marriage because of my illness.” (WATB, Pakistan)* |
| **6. Coping** | | |
|  | Applied coping strategies for dealing with the disease and overcoming related hardship are shared by men and women | *“I believe that God has other plans for me, and*  *those plans must be for my good. I sincerely accept this trial; I hope I will get closer to God.” (WAL, Indonesia)*  *“I would be an example to him and tell him more about how good the treatment is. I would tell him about how bad I looked and how I to tried to eat good diet.” (MATB, South Africa)* |
| **7. Feedback and Recommendations** | | |
|  | Captures suggestions for improving TB and leprosy services, including recommended changes and implementation approaches, with input from both men and women. | *“What I’m trying to say is that some women are not given good food by their in-laws, someone’s parents are too poor, and for TB, food is the most important. So, in this case, if the Government helps, like buying some food-stuffs once a month, or something like that, then that will be a big help.” (WATB, India)*  *“We feel that the government should provide and stiffen laws against bosses that subject their workers to maltreatment so that if they are found ill-treating the workers they should be punished. Also, the government should ensure that all workers are on medical scheme like MASM, and it should be compulsory that each and every worker should be under a medical scheme.” (MATB, Malawi)* |
